# Ultra-small quercetin-based nanotherapeutics ameliorate acute liver failure by combatting inflammation/cellular senescence cycle

**DOI:** 10.7150/thno.103746

**Published:** 2025-01-01

**Authors:** Yali Feng, Xiaoli Zhang, Juan Li, Shan Fu, Weicheng Xu, Jinfeng Liu, Yuan Yang, Tianyan Chen, Yingren Zhao, Dongmin Li, Mingzhen Zhang, Yingli He

**Affiliations:** 1 Department of Infectious Diseases, the First Affiliated Hospital of Xi'an Jiaotong University, Xi'an, Shaanxi, 710061, China.; 2 Shaanxi Clinical Medical Research Center of Infectious Diseases, Xi'an, Shaanxi, 710061, China.; 3 National Regional Infectious Diseases Center Co-constructed by National Health Commission of PRC and People's Government of Shaanxi Province, Xi'an, China.; 4 School of Basic Medical Sciences, Xi'an Jiaotong University, Xi'an, Shaanxi, 710061, China.

**Keywords:** Acute liver failure, Cellular senescence, Inflammation, Quercetin, Quercetin-Fe nanoparticles

## Abstract

**Background:** Acute liver failure (ALF) is marked by a substantial generation of reactive oxygen species (ROS), which can induce both cellular senescence and a pronounced inflammatory response. Senescent cells secrete factors collectively termed the senescence-associated secretory phenotype (SASP), which exacerbate inflammation, while inflammation can reciprocally promote cellular senescence. Quercetin (Que), recognized for its ROS-scavenging capabilities, holds the potential for anti-inflammatory and anti-senescent effects. However, its extremely low aqueous solubility constrains its clinical efficacy in treating inflammation.

**Methods:** We employed a simple and stable coordination method to synthesize ultra-small quercetin-Fe nanoparticles (QFN) by complexing quercetin with iron ions. The ROS-scavenging, anti-inflammatory, and anti-senescent effects of QFN were evaluated *in vitro*. A lipopolysaccharide (LPS)/D-galactosamine (D-GalN)-induced ALF mice model was used to investigate the therapeutic effects of QFN *in vivo*, and transcriptomic analysis was conducted to elucidate the mechanisms underlying QFN-mediated hepatoprotection.

**Results:** Our findings demonstrate that QFN possesses remarkable water solubility and highly efficient ROS-scavenging properties. *In vitro*, QFN effectively inhibits macrophage-mediated inflammation and mitigates hepatocyte senescence. *In vivo*, QFN significantly attenuates LPS/D-GalN-induced ALF by protecting against macrophage inflammation and cellular senescence, thereby disrupting the self-perpetuating cycle of inflammation and aging. Moreover, its potent ROS scavenging capacity not only suppresses cellular apoptosis but also facilitates liver regeneration. Transcriptomic analyses further reveal that QFN exerts its protective effects through the modulation of key pathways involved in cellular senescence and inflammation.

**Conclusions:** In summary, our study characterizes QFN as a potent ROS-scavenging modulator that exhibits both anti-inflammatory and anti-senescent properties, effectively disrupting the detrimental feedback loop between inflammation and cellular senescence. QFN holds considerable potential as a therapeutic agent for the treatment of ALF and other pathologies associated with inflammation and aging.

## Introduction

Acute liver failure (ALF) is a severe and potentially fatal disorder characterized by a rapid decline in liver function, marked by increased reactive oxygen species (ROS) production, elevated inflammatory cytokines, massive hepatocyte death, cellular senescence, and subsequent impaired liver regeneration 1. The overproduction of ROS has been recognized as a key pathological feature strongly linked to the initiation and development of ALF 2. ROS stimulate hepatic macrophages to generate inflammatory cytokines, such as IL-1β, IL-6, and TNF-α, which exacerbate cellular damage and further promote ROS generation 3. The resulting massive ROS production leads to mitochondrial dysfunction, driving cellular senescence or, more seriously, directly inducing apoptosis 4-6. Furthermore, hepatocyte senescence is a key factor hindering liver regeneration, which directly impacts the survival rate in ALF 7. Senescent cells exhibit several key features, including DNA damage, cell cycle arrest, mitochondrial impairment, compromised nuclear membrane integrity, and the secretion of pro-inflammatory molecules, collectively referred to as the senescence-associated secretory phenotype (SASP) 8. SASP, comprising interleukins, cytokines, chemokines, growth factors, and other signaling molecules, can propagate senescent signals to neighboring hepatocytes, thereby impairing liver regeneration capacity during ALF 9. Moreover, SASP further promotes macrophage activation and amplifies inflammation, creating a vicious cycle between inflammation and cellular senescence 7, 10. Therefore, limiting ROS, which can alleviate both inflammation and senescence, is recognized as a crucial approach for mitigating ALF. Consequently, there is an urgent need to develop drugs with strong ROS scavenging effects to break this cycle and alleviate liver damage.

Quercetin, a natural polyphenol found abundantly in various fruits and vegetables, is widely recognized for its antioxidant and anti-inflammatory properties, making it a promising candidate for therapies targeting ROS-related diseases 11, 12. Previous studies have demonstrated that oral administration of quercetin in mice can mitigate ALF by modulating ferroptosis and mitochondria-mediated apoptosis 13, 14. Notably, the combination of quercetin with dasatinib, a tyrosine kinase inhibitor, has been identified as one of the first senolytic agents, though its precise senolytic mechanism remains unclear 15, 16. Given the potential risks associated with dasatinib, including pleural effusion, pulmonary arterial hypertension, and hematological toxicity 17, 18, quercetin has also been explored as an independent anti-aging intervention due to its strong antioxidant properties 19. Studies have shown that quercetin treatment can mitigate ovarian aging, reduce skin aging, and promote hair regrowth 20-22. However, due to the extremely poor water solubility of quercetin, current *in vivo* studies have relied on large oral doses, typically ranging from 10 to 50 mg/kg body weight 15, 16, 20. Furthermore, suboptimal absorption, low oral bioavailability, and significant inter-individual variability in absorption and metabolism present challenges to achieving consistent therapeutic outcomes and complicate the optimization of clinical dosing.

To address these limitations, significant efforts have been devoted to modifying the properties of quercetin to enhance its biological activity, improve its solubility, and increase its bioavailability 13, 14, 23. In an oxidative stress-induced senescent cell model, quercetin was functionalized with Fe_3_O_4_ nanoparticles, which delayed cellular senescence by promoting AMPK activity 14. Solid lipid nanoparticles have also been employed for the targeted delivery of quercetin in the treatment of age-related macular degeneration 23. In a previous study, Bolin Bao *et al.* functionalized quercetin with copper (Cu) and selenium (Se) to form ultra-small nanoparticles, which effectively scavenged ROS and increased the expression of intracellular antioxidative enzymes, significantly reducing cellular oxidative stress and protecting against acute kidney injury 13. Moreover, the self-assembly of metal-polyphenol complexes has been explored as a versatile platform to enhance both the solubility and bioavailability of these compounds 24, 25.

**Scheme 1:**
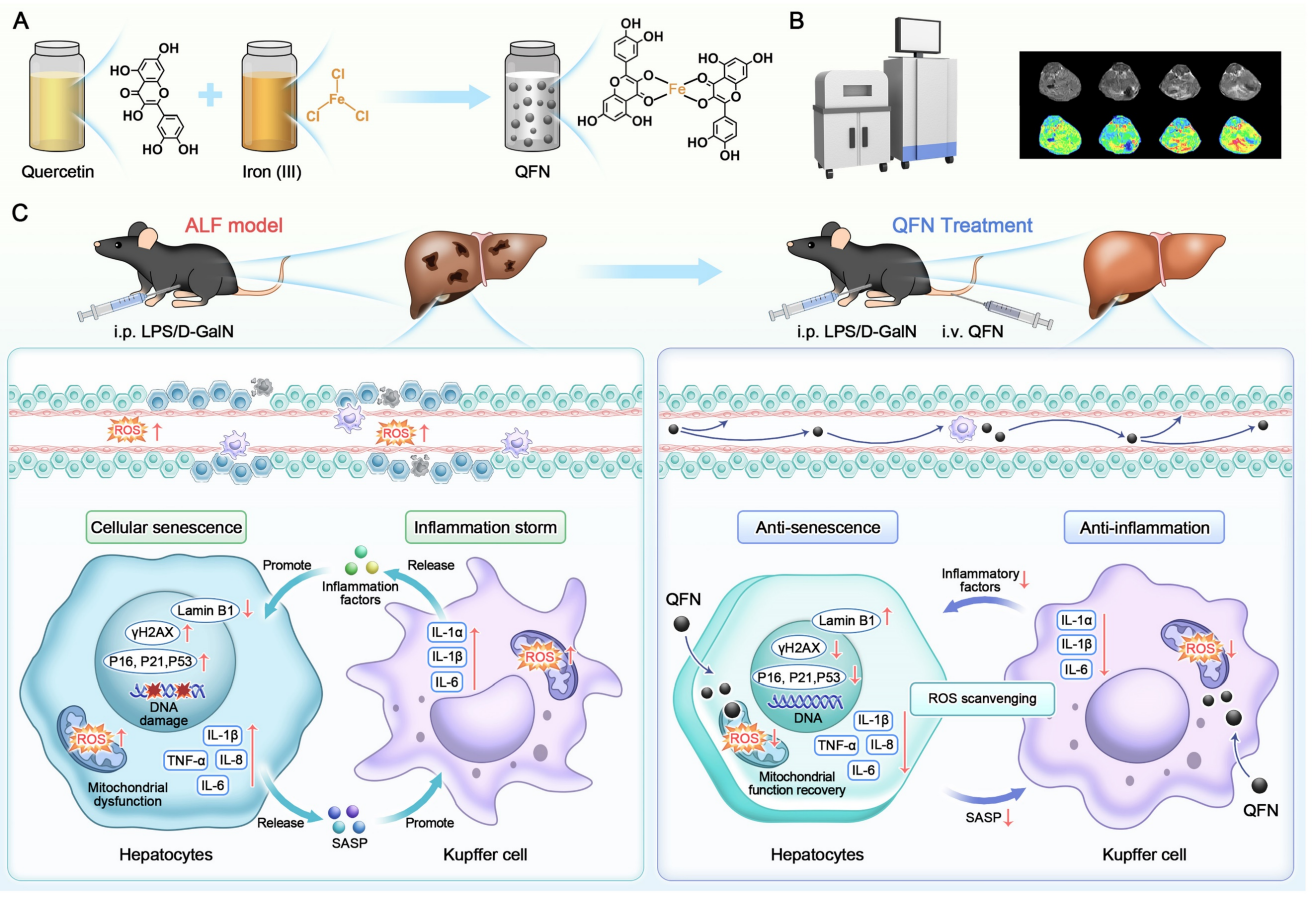
** Schematic illustration of QFN preparation and its anti-inflammatory and anti-senescent effects in protecting against ALF. (A)** The synthetic process of QFN. **(B)** T1-weighted MRI of QFN in mice. **(C)** Acute liver failure, induced by co-administration of LPS/D-GalN, is characterized by massive cell death, cellular senescence, and an inflammatory storm. QFN was intravenously injected into ALF mice and efficiently protected against ALF through its ROS scavenging ability. Additionally, QFN inhibited the inflammatory response, reduced cellular senescence, and broke the vicious cycle between inflammation and aging, further promoting liver repair and regeneration.

In this study, we synthesized the ultra-small auto-coordinated nanoparticles, QFN, by mixing Fe^3+^ with the natural antioxidant quercetin, which can significantly improve its low water solubility and poor bioavailability. Iron has a broader safety margin and a lower risk of toxicity compared to other metallic elements. Furthermore, the inclusion of Fe^3+^ enables QFN to be utilized in MRI T1 imaging, facilitating the detection of QFN accumulation in the liver and the assessment of liver necrosis. QFN exhibited a strong ability to scavenge broad-spectrum ROS and protect against mitochondrial dysfunction, thereby effectively reducing inflammatory responses and counteracting cellular senescence. More significantly, QFN demonstrated a substantial hepatoprotective effect in LPS/D-GalN-induced ALF mice, coupled with a favorable safety profile. Transcriptomic analysis was employed to further explore the underlying mechanisms, revealing that QFN administration significantly alleviated macrophage-driven inflammation, reduced hepatocyte apoptosis, inhibited cellular senescence and SASP secretion, and promoted liver regeneration, thereby protecting against ALF **(Scheme 1)**. Our study introduces a novel therapeutic approach to disrupting the cycle of reciprocal reinforcement between inflammation and cellular aging. Additionally, it provides innovative strategies for the *in vivo* delivery of quercetin with enhanced safety, paving the way for further clinical translation and application.

## Material and methods

### Synthesis of QFN

First, 20 mg of FeCl_3_•6H_2_O was dissolved in 1 mL of methanol and gradually introduced to a solution of 66 mg of polyvinyl pyrrolidone (PVP) in 5 mL of methanol. The resulting mixture was continuously stirred for 5 min. Separately, 10 mg of quercetin dihydrate was dissolved in 1 mL of methanol and sonicated for 10 min. The quercetin solution was then gradually added to the FeCl_3_-PVP mixture, which was stirred continuously for 3 h. Following the stirring process, the solution was moved to a 3500-Da MWCO dialysis bag and subjected to dialysis in deionized water for 24 h to remove methanol and any excess substances 26. All reactions were performed at room temperature. The final product, the QFN complex coordinated by Fe and quercetin, was collected and stored at 4°C for subsequent use.

### Synthesis of QFN-Cy5.5

First, QFN was treated with ethylenediamine (EDA) to introduce amino groups and obtain the amine-functionalized QFN (QFN-NH_2_). Dialyze the reaction mixture overnight against deionized water to remove any unreacted EDA. The purified QFN-NH_2_ (5 mg/mL) is then reacted with the carboxyl group of the reactive dye Cy5.5-NHS ester (0.2 mg/mL) in phosphate buffer at room temperature. The reaction mixture is stirred in the dark for 24 h to facilitate coupling via amide bond formation. The resulting QFN-Cy5.5 mixture is purified using a dialysis bag (MWCO: 3500 Da) and dialyzed against deionized water for 24 h to remove unreacted Cy5.5. Finally, collect the conjugated product and confirm the successful attachment of Cy5.5 to QFN using fluorescence spectroscopy.

### Cell culture

The RAW264.7 murine macrophage cell line and the HepG2 human hepatoma cell line were maintained in a DMEM medium containing 10% fetal bovine serum (FBS) and 1% penicillin-streptomycin for supplementation. The AML12 mouse hepatocyte cell line was maintained in DMEM medium enriched with 10% FBS, 1% insulin-transferrin-selenium (ITS), and 40 ng/mL dexamethasone. All cell cultures were incubated at 37 °C in a humidified environment with 5% CO_2_.

### Cellular uptake

QFN was labeled with Cy5.5 (near-infrared fluorescent dye). AML12 cells were treated with QFN-Cy5.5 (10, 20, and 40 μg/mL) for 6 h, or with 20 μg/mL QFN-Cy5.5 for different incubation times (4 h, 6 h, and 8 h). After incubation, cellular uptake of QFN-Cy5.5 was evaluated using fluorescence imaging (Carl Zeiss, Germany) and flow cytometry (Becton Dickinson, USA). Actin filaments were stained with Actin-Tracker Green (C2201S, Beyotime, China), and nuclei were stained with DAPI (C1005, Beyotime, China).

To examine the cellular uptake mechanism of QFN in AML12 cells, different endocytosis inhibitors were employed. Specifically, amiloride (Ami), chlorpromazine (CPZ), and methyl-β-cyclodextrin (MβCD) were introduced into the medium at concentrations of 100 µM, 100 µM, and 40 µM, respectively. AML12 cells were pretreated with these inhibitors at 37 ºC for 1 h, followed by incubation with 20 µg/mL QFN-Cy5.5 for 6 h. Cellular uptake was then assessed using fluorescence imaging and flow cytometry.

### *In vitro* cytoprotective study of QFN

AML12 cells were treated with 200 μM H_2_O_2_ for 2 h to induce cell apoptosis and then incubated with 5, 10, 20, 40, and 80 μg/mL QDN for 24 h to assess cell viability using the CCK-8 method. For apoptosis assessment, AML12 cells were exposed to 200 μM H_2_O_2_ for 2 h to induce apoptosis, followed by co-incubation with either 10 µg/mL quercetin or 10 µg/mL QFN for 24 h. Cell apoptosis was then evaluated using the Calcein-AM and PI (PF00007, Proteintech, China) through immunofluorescent staining.

### *In vitro* ROS scavenging ability of QFN

Dichlorodihydrofluorescein diacetate (DCFH-DA) and dihydroethidium (DHE) were used for ROS detection. In brief, RAW264.7 cells were cultured overnight, pretreated with 10 µg/mL quercetin or 10 µg/mL QFN for 6 h, and then incubated with 800 µM H_2_O_2_ for 3 h to induce oxidative stress. Subsequently, they were incubated with 10 µM DCFH-DA and 5 µM DHE at 37 °C for 30 min away from light. After washing with phosphate-buffered saline (PBS), the cells were assessed ROS levels using a fluorescence microscope and flow cytometry. In AML12 cells, ROS and oxidative stress were induced by incubation with 50 µM H_2_O_2_ for 2 h to evaluate the ROS scavenging ability of QFN.

### Anti-inflammatory effect of QFN in Raw264.7 cells

RAW264.7 cells were pretreated with 10 µg/mL quercetin or 10 µg/mL QFN for 6 h, followed by incubation with 200 ng/mL lipopolysaccharide (LPS) for 12 h. After incubation, the inhibitory effect of QFN on LPS-induced M1 polarization of RAW264.7 cells was evaluated by immunofluorescence staining using an antibody against CD86 (13395-1-AP, Proteintech, China) and flow cytometry with APC Rat anti-Mouse CD86 (561964, BD Bioscience, USA). Additionally, the gene expression of inflammation-related cytokines in RAW264.7 cells was detected using qPCR. The primer sequences of related genes are shown in Table S1.

### Anti-senescent effect of QFN in hepatocytes

AML12 cells were treated with 4 μM etoposide (ETO, HY-13629, MedChemExpress, USA) for 24 h, followed by incubation in ETO-free complete conditioned media for an additional 24 h to induce cellular senescence. During the entire period, 10 µg/mL quercetin or 10 µg/mL QFN was added. The cells were then collected for immunofluorescence staining, Western blotting, and qPCR to evaluate cellular senescence.

HepG2 cells were treated with 300 μM H_2_O_2_ for 1 h and then cultured in an H_2_O_2_-free medium for 3 days to induce senescence. Quercetin and QFN were added during the recovery period until the cells were collected.

### Construction of ALF Model and QFN treatment

Male C57BL/6J mice (6-8 weeks old, 18-20 g) were procured from GemPharmatech Co., Ltd (Jiangsu, China). All animal experimental procedures and welfare adhered to the standards established by the Institutional Animal Care Committee of Xi'an Jiaotong University and were approved by the Ethics Committee of the First Affiliated Hospital of Xi'an Jiaotong University. To induce ALF in mice, LPS (30 μg/kg body weight, Sigma-Aldrich, USA) and D-GalN (300 mg/kg body weight, Psaitong, China) were administered intraperitoneally in PBS. To evaluate the protective efficacy of QFN against ALF, C57BL/6J mice received intravenous injections of QFN at varying doses (high: 10 mg/kg body weight; medium: 5 mg/kg body weight; low: 2.5 mg/kg body weight) at two time points: 24 h before and concurrently with LPS/D-GalN administration. Mice were euthanized 6 h after the administration of LPS/D-GalN injections, and blood samples and liver tissues were collected for subsequent experiments.

### Transcriptomic sequencing

Mice livers were frozen in liquid nitrogen and sent to Lc-Bio Technologies (Hangzhou, China) for total RNA extraction and genome-wide transcriptomic analysis. Genes with differential expression were identified using a fold change > 2 and a P value < 0.05 as criteria. Gene Ontology (GO), the Kyoto Encyclopedia of Genes and Genomes (KEGG), and Gene Set Enrichment Analysis (GSEA) enrichment studies were carried out according to the method provided by Lc-Bio (https://www.omicstudio.cn/home).

### Network construction and analysis

Quercetin-related targets were obtained from the Traditional Chinese Medicine Systems Pharmacology (TCMSP) database (https://www.tcmsp-e.com). The GeneCards database (https://www.genecards.org) was searched using “acute liver failure” as the keyword. Common targets of quercetin and ALF were identified and visualized using a Venn diagram generated by OmicStudio tools. The protein-protein interaction (PPI) network was constructed by the shared genes using the STRING database (https://string-db.org) and visualized using Cytoscape 3.6.1. KEGG enrichment pathway analyses were performed with OmicStudio tools (https://www.omicstudio.cn/tool) to identify pathways associated with the key targets.

### Data and statistical analysis

Data were presented as the mean ± SEM and data analysis was conducted using GraphPad Prism version 8.0. For comparisons between two groups, an unpaired two-tailed Student's t-test was applied, while for multiple-group comparisons, a one-way ANOVA was used. Statistical significance was set at P < 0.05 (ns: not significant, *P < 0.05, **P < 0.01, ***P < 0.001, ****P < 0.0001).

## Results and Discussion

### ALF is closely related to cellular senescence and inflammatory response

**Figure 1:**
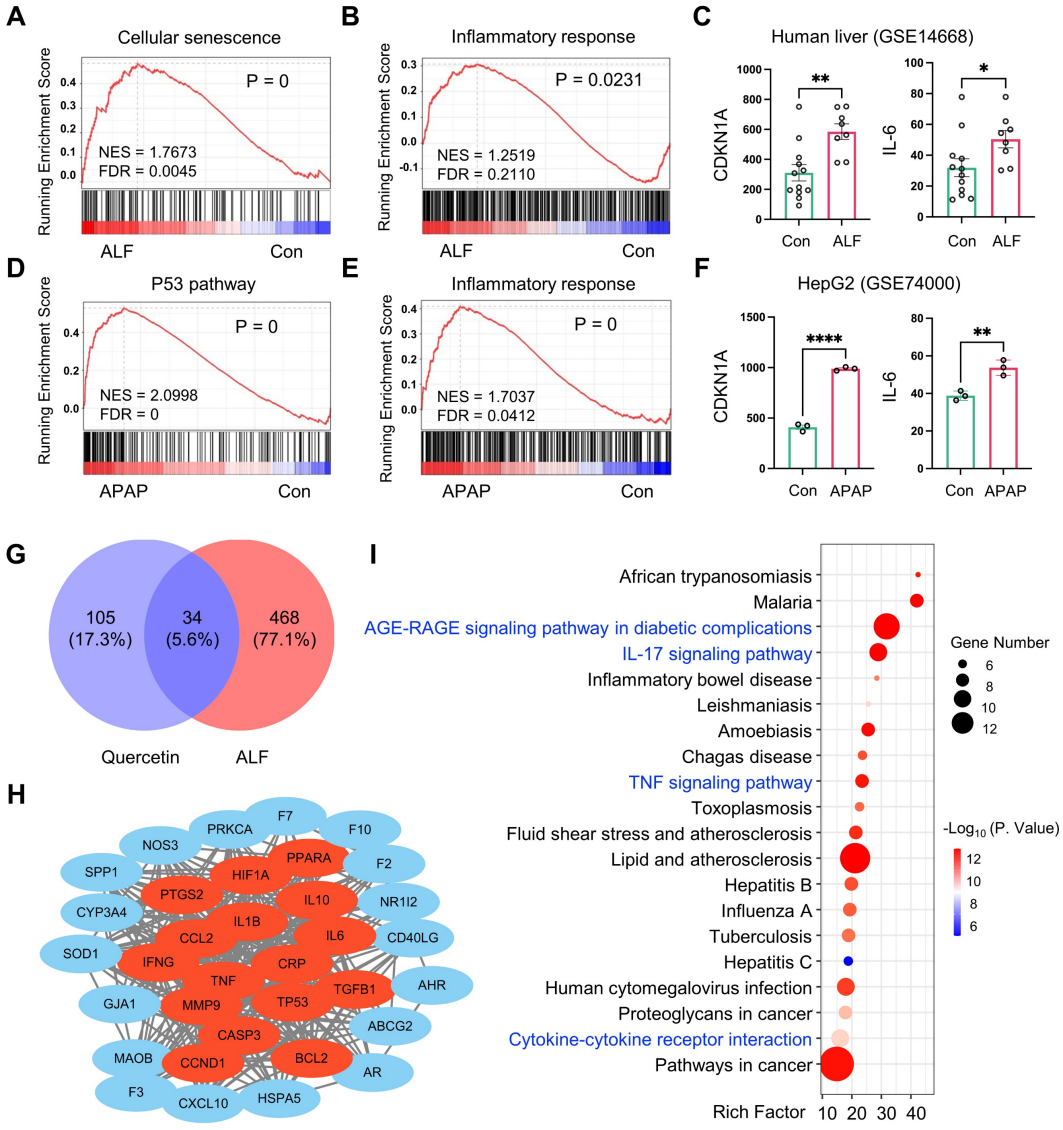
** ALF exhibits cellular senescence and inflammatory response.** Gene Set Enrichment Analysis (GSEA) of cellular senescence **(A)** and inflammatory response **(B)** using RNA-seq data derived from liver samples of a human acute liver failure (ALF) cohort (GEO accession no. GSE14668). **(C)** Gene expression of CDKN1A and IL-6 in human ALF liver samples (n = 12 in the control group, n = 8 in the ALF group, GEO accession no. GSE14668). GSEA of genes related to P53 signaling pathway **(D)** and inflammatory response **(E)** in acetaminophen (APAP) exposure HepG2 cells (GEO accession no. GSE74000). **(F)** Gene expression of *CDKN1A* and *IL-6* in APAP-exposed HepG2 cells (n = 3 in control and APAP group, GEO accession no. GSE74000). **(G)** Venn diagram of shared quercetin and ALF-related targets. **(H)** Visualization of the PPI network by Cytoscape 3.9.1. Data are expressed as mean ± SEM. *P < 0.05, **P < 0.01, ****P < 0.0001 (unpaired t-test). **(I)** Top 20 KEGG pathways of the shared genes from quercetin and ALF (p < 0.05).

Aging, characterized by cellular senescence, is recognized as a major factor in the development of chronic liver disease 27, 28. However, its role in acute liver disease, including ALF, remains poorly understood. To investigate whether cellular senescence is associated with ALF, we performed GSEA analysis using RNA sequencing (RNA-seq) data obtained from the Gene Expression Omnibus (GEO). We analyzed bulk liver RNA-seq datasets from patients with HBV-associated ALF and those who underwent resection for angioma (GEO accession no. GSE14668). The GSEA results revealed significant enrichment of genes related to cellular senescence and inflammatory responses in ALF livers (**Figure 1A-B**). Furthermore, we analyzed the gene expression of the cell cycle arrest marker *CDKN1A* and the pro-inflammatory cytokine *IL-6* in liver samples from this cohort, and both were significantly overexpressed in ALF individuals (**Figure 1C**). We also analyzed a publicly available dataset (GEO accession no. GSE74000) to compare the transcriptomic signatures of HepG2 cells (a human liver cancer cell line) exposed to acetaminophen (APAP). The GSEA of this dataset revealed a significant enrichment of genes associated with the p53 signaling pathway and the inflammatory response in hepatic cells with APAP-induced injury (**Figure 1D-E**). Additionally, the gene expression of *CDKN1A* and *IL-6* was significantly upregulated in hepatocytes following APAP stimulation (**Figure 1F**). Taken together, these findings suggest that cellular senescence and the related inflammatory response may play a role in the progression of ALF, indicating potential targets for therapeutic intervention.

Quercetin is well-known for its antioxidant and anti-inflammatory properties, which are linked to the prevention of aging-related diseases 29. However, its effect on ALF is not fully understood. This issue was investigated using a network pharmacology strategy. Quercetin's potential target genes were retrieved from the TCMSP database. A search using “acute liver failure” as a keyword in the GeneCards database yielded 502 genes related to ALF. A Venn diagram identified 34 overlapping targets between quercetin and ALF (**Figure 1G**). The PPI network was constructed using overlapping genes (**Figure S1**) and visualized for protein interaction strength using the CytoNCA plugin in Cytoscape 3.9.1. Larger, more deeply colored nodes closer to the center indicated higher degree values, signifying stronger interactions among the targets. The top 5 core targets were identified: IL6, TNF, IL1B, CASP3, and TP53 (**Figure 1H**), all of which are strongly associated with senescence and the SASP. To further investigate the protective mechanism of quercetin in ALF, we performed the KEGG enrichment analysis using OmicStudio tools. The overlapping cluster of quercetin and ALF targets was enriched in genes and pathways related to the AGE-RAGE signaling pathway in diabetic complications, the IL-17 signaling pathway, the TNF signaling pathway, and cytokine-cytokine receptor interaction, all of which are involved in the mechanisms of cellular senescence and inflammation (**Figure 1I**). These findings confirm that the connection between quercetin and ALF is related to cellular senescence. Therefore, it is hypothesized that quercetin may represent a promising strategy for the prevention and treatment of ALF by modulating cellular senescence and inflammation.

### Construction of the QFN and physicochemical characterization

**Figure 2:**
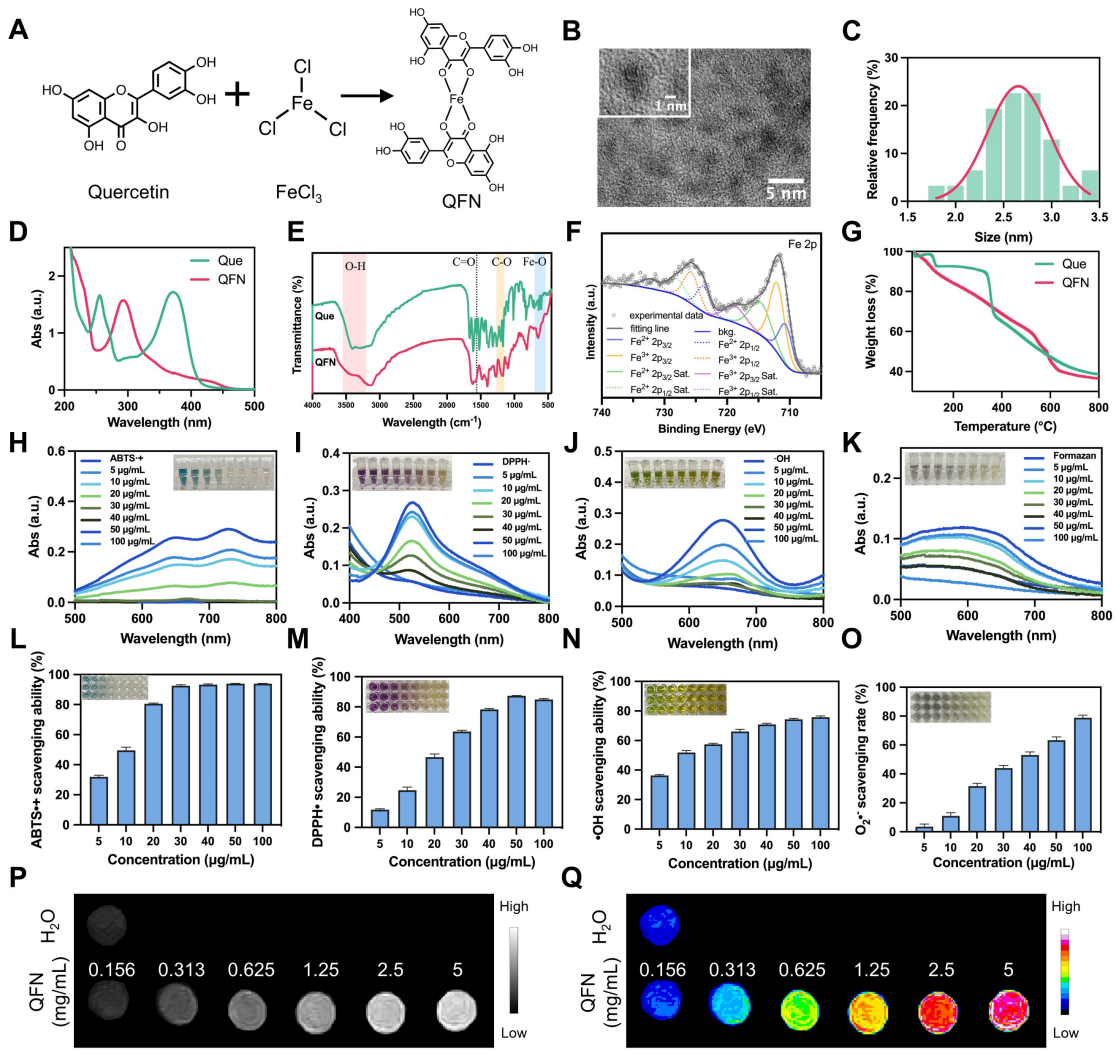
** Synthesis and characterization of QFN. (A)** Schematic illustration of the synthetic process of QFN. **(B)** TEM image of the synthesized QFN. Scale bar = 5 nm. **(C)** Nanodot sizes of QFN were observed under TEM. **(D)** UV-Vis spectra of quercetin and QFN in methanol. **(E)** FT-IR spectra of quercetin and QFN. **(F)** XPS spectra of Fe in QFN. **(G)** Thermogravimetric analysis (TGA) of quercetin and QFN. **(H-K)** UV-Vis absorbance spectra of ABTS•+ radicals **(H)**, DPPH• radicals **(I)**, hydroxyl radicals **(J)**, and formazan **(K)** after incubation with various concentrations of QFN. **(L-O)** Quantitative analysis of ABTS•+ scavenging ability **(L)**, DPPH• scavenging ability **(M)**, hydroxyl radicals ability **(N)**, and superoxide anion scavenging rate **(O)** at various concentrations of QFN. **(P)** T1-weighted MRI of QFN at different concentrations. **(Q)** Pseudo-color maps reconstructed from the signal intensity of T1-weighted MRI.

The clinical use of quercetin is limited by its significantly low water solubility 30. To overcome this challenge, ultra-small quercetin-Fe natural coordination nanoparticles (QFN) were synthesized using a straightforward and stable method (**Figure 2A**). The successful coordination of ferric ions with the phenolic groups of quercetin was indicated by a color change from yellow to deep dark. The obtained QFN significantly improved the solubility of quercetin in water **(Figure S2A)**. Transmission electron microscopy (TEM) analysis showed that QFN particles were ultra-small, with sizes ranging from 2-3 nm **(Figure 2B-C)**. The zeta potential of QFN was measured at approximately -17.99 mV** (Figure S2B)**. Unlike quercetin molecules, which exhibit two distinct absorption peaks at 265 nm and 370 nm, QFN displays a single absorption peak at 293 nm in the UV-Vis spectrum (**Figure 2D**). This peaks suggest charge transfer interactions between the ligand and the metal, further confirming the successful formation of QFNs with good purity. The monitoring of the polydispersity index (PDI) and UV-Vis absorption spectra of QFN over time demonstrated its long-term stability in aqueous suspension **(Figure S2C-D)**.

Fourier transform infrared spectroscopy (FT-IR) analysis revealed the formation of new Fe-O bonds, as indicated by stretching observed in the 540-700 cm^-1^ range. The O-H stretching band (3200-3550 cm^-1^) showed reduced intensity, while the hydroxyl C-O stretching band (1150-1270 cm^-1^) became a broad shoulder. Additionally, the C=O stretching band around 1560 cm^-1^ exhibited a noticeable decrease in intensity, suggesting the involvement of the C=O group in binding **(Figure 2E)**. These findings indicate the coordination of Fe^3+^ ions with both the phenolic hydroxyl and ketone groups in quercetin. X-ray photoelectron spectroscopy (XPS) analysis provided detailed insights into the chemical bonds present in QFN, with peak fitting observed for C 1s, O 1s, and Fe 2p **(Figure S3A)**. In the C 1s spectrum, the observed binding energy peaks were ascribed to the presence of C=C, C-C, C-O, and C=O bonds **(Figure S3B)**. Furthermore, analysis of the O 1s spectrum provided evidence of metal-O bonds **(Figure S3C)**. Moreover, the Fe 2p spectrum indicated the coexistence of Fe^2+^ and Fe^3+^ ions in QFN, with the Fe^2+^/Fe^3+^ ratio being approximately 1:3 **(Figure 2F)**. The mass of the QFN in this research was determined by weighing the freeze-dried particles. Meanwhile, the iron concentration in the QFN was measured at 76.11 mg/L using inductively coupled plasma optical emission spectrometry (ICP-OES), which corresponds to 1.36 mM based on the molecular mass of iron. Thermogravimetric analysis (TGA) revealed a molar ratio of quercetin to ferric ions of 2:1, indicating that each iron ion coordinates with two quercetin molecules (**Figure 2G**). Together with FT-IR results, these findings suggest that QFN forms a complex in which the central Fe atom is coordinated with four oxygen atoms from two five-membered rings of quercetin (**Figure 2A**). Consistent with previous studies 26, 31, these findings suggest that QFN not only overcome the solubility limitations of quercetin but also enhance its stability and functionality, making it a promising candidate for clinical applications.

Since quercetin is commonly recognized as a natural antioxidant, we initially assessed the antioxidant capacity of QFN *in vitro*. Various probes, including ABTS, DPPH, TMB, and NBT, are widely used to explore the ROS scavenging abilities of nanomaterials. The ABTS assay, in which ABTS is oxidized by potassium persulfate (K_2_S_2_O_8_) to produce a blue-green cationic radical (ABTS•+), was used to evaluate the antioxidant effect of QFN. In the presence of QFN, the solution's color gradually faded from green to colorless with increasing concentrations of QFN (**Figure 2H**). Correspondingly, the characteristic absorption of ABTS at 734 nm significantly decreased, with over 90% of ABTS•+ radicals being neutralized at low QFN concentrations (**Figure 2L**). The results of the ABTS assay suggest that QFN possesses strong total antioxidant capacity. However, to definitively confirm this, antioxidant capacity in different solvent systems (such as anhydrous ethanol), as assessed by the DPPH assay, is required. Consistent with the ABTS findings, DPPH radicals, characterized by a distinct absorption peak at 517 nm, also showed a reduction in absorbance upon interaction with QFN. This indicates that QFN demonstrates a concentration-dependent scavenging effect on DPPH radicals **(Figure 2I and 2M)**. Furthermore, the scavenging activity against hydroxyl radicals (•OH) was evaluated using the TMB assay. As the concentration of QFN increased, its ability to scavenge •OH radicals also increased. Notably, even at a concentration of 10 μg/mL, QFN was able to eliminate more than 50% of hydroxyl radicals **(Figure 2J and 2N)**. The NBT assay specifically quantifies the removal of superoxide anions (O_2_•^-^) by QFN. The reduction in UV absorbance of the converted NBT (formazan) at 560 nm indicated that the scavenging efficiency of QFN against O_2_•^-^ was also concentration-dependent, demonstrating its superoxide dismutase (SOD)-mimetic properties **(Figure 2K and 2O)**. SOD is a widely distributed and stable antioxidant enzyme that catalyzes the conversion of O_2_•^-^ into H_2_O_2_ and O_2_. The SOD-like activity of QFN was further confirmed using the water-soluble tetrazolium (WST) assay, which revealed a SOD-like activity value of 40.2 U/mg **(Figure S4)**. Taken together, these results strongly indicate that QFN, owing to the presence of phenolic and ketone groups in the quercetin structure, possesses robust ROS scavenging abilities, making it a promising antioxidant for treating oxidative stress-related diseases.

Fe^3+^-based compounds have demonstrated the potential to enhance T1-weighted magnetic resonance imaging (MRI), which offers high-resolution images and is particularly beneficial for disease diagnosis and treatment 32-34. In this study, we assess the effect of QFN on T1-MRI performance. The analysis of the resulting T1-weighted images revealed that the T1 signal of QFN was significantly stronger than that of H_2_O, with further signal enhancement observed as nanoparticle concentration increased **(Figure 2P-Q)**. These findings confirm the strong potential of QFN as a T1 MRI contrast agent for disease diagnosis.

### QFN is primarily internalized by cells through clathrin-mediated endocytosis

**Figure 3:**
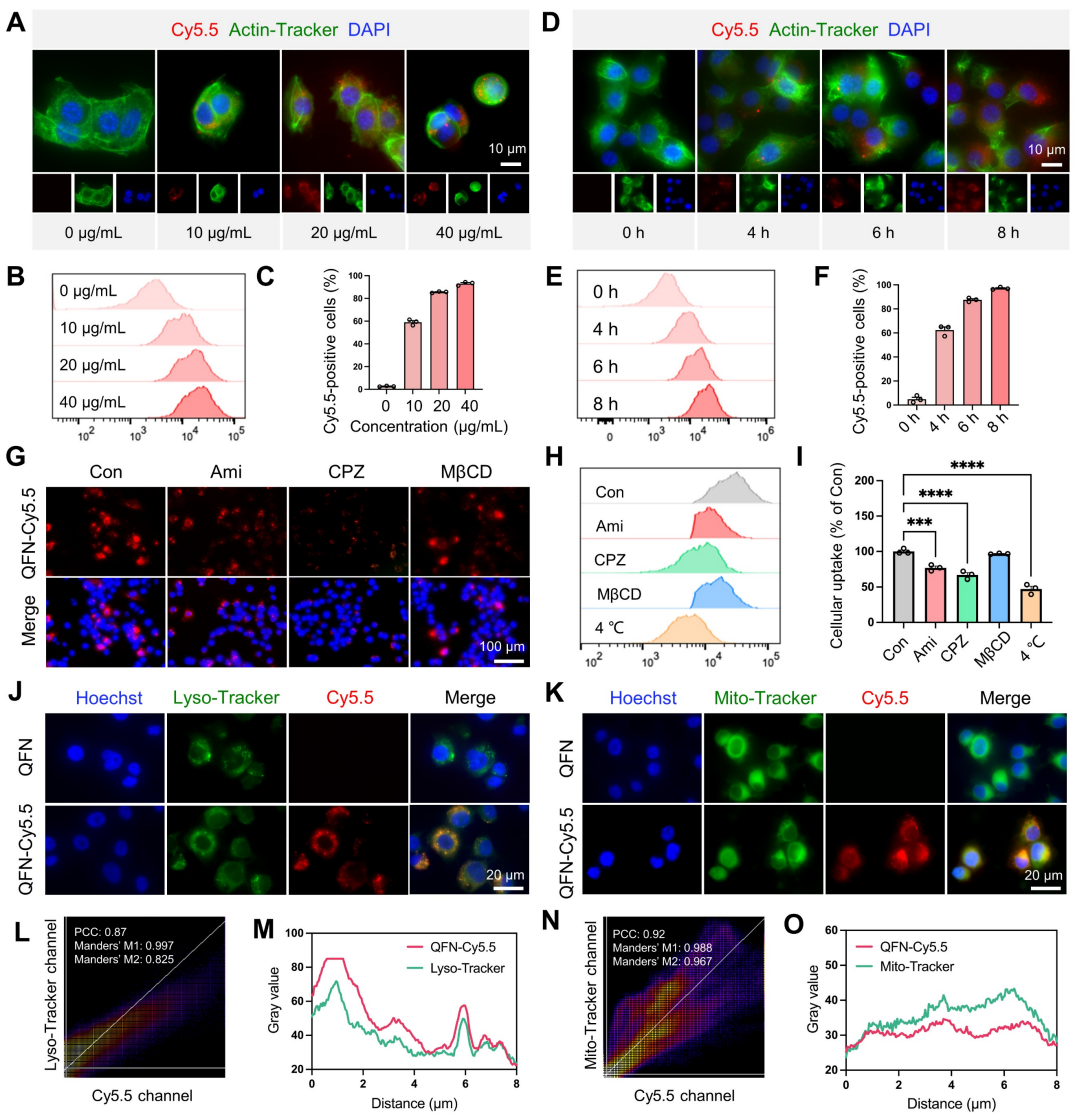
** Cellular uptake and intracellular distribution of QFN.** Representative images **(A)** and flow cytometry analysis **(B)** of AML12 cells incubated with different concentrations of QFN-Cy5.5 for 6 h. Scale bar = 10μm. **(C)** Percentage of Cy5.5-positive AML12 cells from (B). Representative images **(D)** and flow cytometry analysis **(E)** of AML12 cells incubated with 20 μg/mL QFN-Cy5.5 for different periods. Scale bar = 10 μm. **(F)** Percentage of Cy5.5-positive AML12 cells from (E). **(G-I)** AML12 cells were cooled to 4 °C or separately pretreated with endocytosis-related inhibitors at 37 °C for 1 h, followed by incubation with 20 μg/mL QFN-Cy5.5 for 6 h. Ami, amiloride; CPZ, chlorpromazine; MβCD, methyl-β-cyclodextrin. Fluorescent imaging **(G)** and flow cytometry analysis **(H)** of QFN-Cy5.5 in AML12 cells. Scale bar = 100 μm. **(I)** Percentage of Cy5.5-positive AML12 cells from (H). **(J-O)** AML12 cells were incubated with 20 μg/mL QFN-Cy5.5 for 6 h. Lysosomes and mitochondria were labeled with Lyso-Tracker Green and Mito-Tracker Green, respectively. Images were captured using a confocal microscope. Co-localization of QFN-Cy5.5 with lysosomes **(J)** and with mitochondria **(K)**. Scale bar = 20 μm. Pearson's correlation coefficient (PCC) and Mander's correlation coefficient (MCC) analyses of QFN-Cy5.5 with lysosomes **(L)** or mitochondria **(N)**, respectively. Plot profile analysis of QFN-Cy5.5 co-localization with Lyso-Tracker **(M)** or Mito-Tracker **(O)**, respectively. Data are presented as mean ± SEM, *P < 0.05, ****P < 0.0001 (one-way ANOVA test with Tukey's multiple comparisons test).

To explore the role of QFN in hepatocytes, we investigated the uptake mechanism of QFN by these cells. Initially, QFN was labeled with the near-infrared fluorescent dye cyanine5.5 (Cy5.5), resulting in the modified QFN-Cy5.5. Fluorescent imaging and flow cytometry analyses demonstrated that QFN internalization by AML12 cells occurred in both a time- and concentration-dependent manner **(Figure 3A-F)**. As the concentration of QFN-Cy5.5 increased, the intracellular fluorescence intensity of Cy5.5 within AML12 cells also rose, indicating that the uptake of QFN-Cy5.5 by AML12 cells was concentration-dependent **(Figure 3A-C)**. Significant uptake was observed after 4 h, with a marked increase reaching over 90% following 6 h of incubation, confirming that QFN accumulation in the cells is time-dependent **(Figure 3D-F)**. The uptake of QFN by hepatocytes is a prerequisite for its subsequent functions within these cells. Next, we investigated the mechanism by which hepatocytes internalize QFN. The nanoparticles primarily entered the cells through membrane fusion and endocytosis. Endocytosis involves various mechanisms, including clathrin-mediated endocytosis, caveolin-mediated endocytosis, and macropinocytosis, among others 35, 36. Using specific inhibitors, we determined that blocking clathrin-mediated endocytosis with chlorpromazine (CPZ) resulted in an approximate 40% reduction in QFN-Cy5.5 uptake, whereas inhibition of caveolae-mediated endocytosis with methyl-β-cyclodextrin (MβCD) had no significant effect. In contrast, exposure to amiloride (Ami), a potent macropinocytosis inhibitor, only slightly reduced QFN-Cy5.5 uptake **(Figure 3G-I)**. Consistent with previous research, our results have shown that the clathrin pathway is considered one of the most important pathways mediating QFN endocytosis 37. Flow cytometry results further revealed that cellular uptake of QFN was inhibited at lower temperatures, suggesting that QFN may be internalized by hepatocytes via a membrane fusion pathway** (Figure 3H-I)**.

To further determine the subcellular localization of QFN-Cy5.5, AML12 cells were incubated with QFN-Cy5.5 for 6 h, followed by staining with Lyso-Tracker and Mito-Tracker to label lysosomes and mitochondria, respectively. Confocal microscopy revealed significant co-localization of QFN-Cy5.5 with both lysosomes and mitochondria **(Figure 3J-K)**. The extent of this co-localization was quantified using Pearson's correlation coefficient (PCC) and Manders' co-localization coefficient (MCC), with the results further supported by plot profile analyses **(Figure 3L-O)**. Mitochondria are the primary organelles responsible for ROS production following injury, which can further promote cellular senescence and inflammation. Therefore, the co-localization of QFN with mitochondria supports the role of QFN in scavenging ROS and protecting against ROS-related diseases.

### ROS scavenging and anti-inflammatory activity of QFN* in vitro*

**Figure 4:**
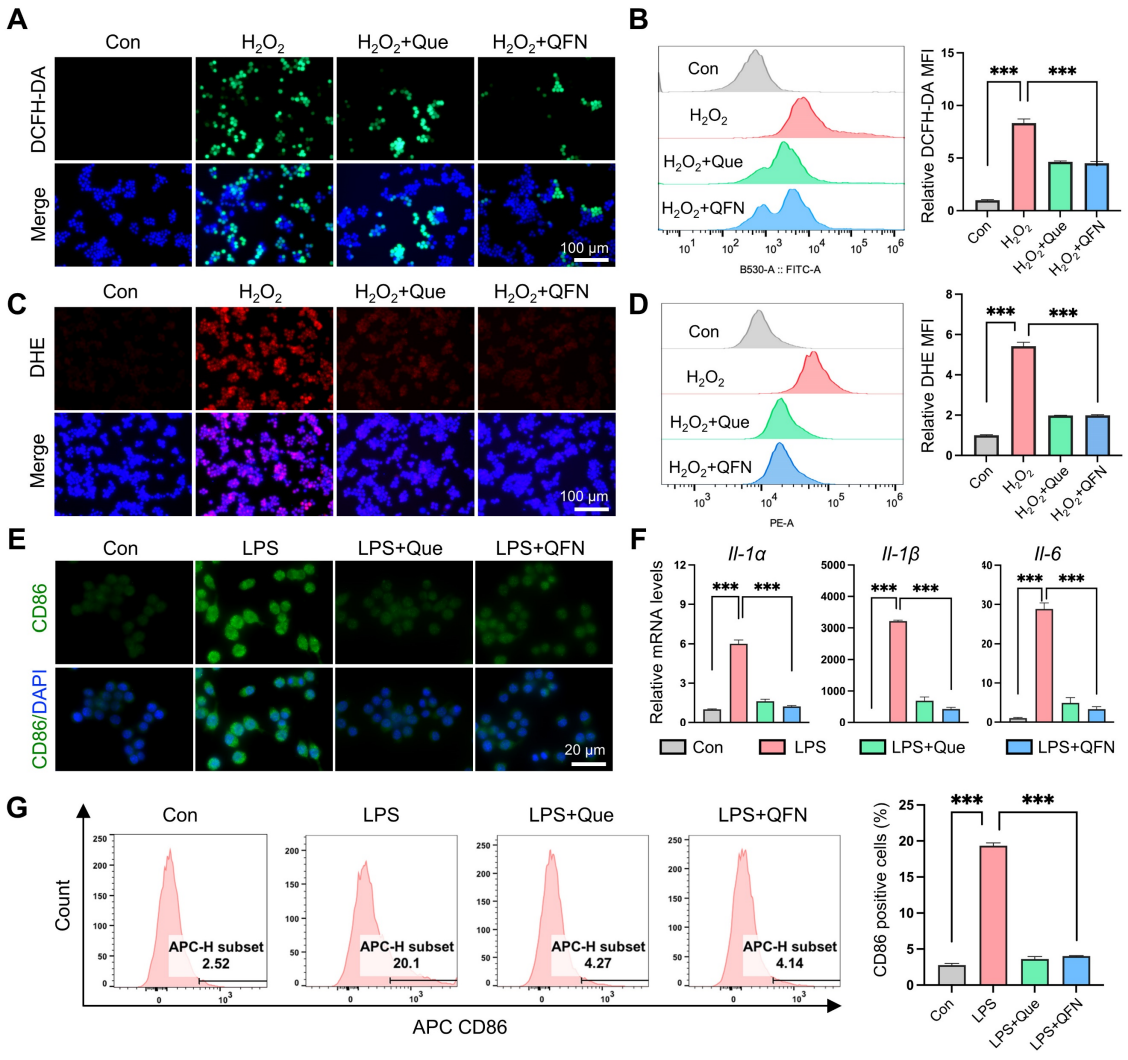
** ROS scavenging and anti-inflammatory ability of QFN *in vitro*. (A-D)** Raw264.7 cells were pretreated with 10 μg/mL quercetin or 10 μg/mL QFN for 6 h then incubated with 800 μm H_2_O_2_ for 3 h. **(A)** Representative images of DCFH-DA. Scale bar = 100μm. **(B)** Flow cytometry (left) of DCFH-DA and the relative MFI (right) in Raw264.7 cells. **(C)** Representative images of DHE. Scale bar = 100μm. **(D)** Flow cytometry (left) of DHE and the relative MFI (right) in Raw264.7 cells. **(E-G)** Raw264.7 cells were pretreated with 10 μg/mL quercetin or 10 μg/mL QFN for 6 h then incubated with 200 ng/mL LPS for 12 h. **(E)** Representative images of immunofluorescence staining for CD86. **(F)** Gene expression of *Il-1α, Il-1β*, and* Il-6*. **(G)** Intracellular CD86 fluorescence was detected by flow cytometry and the quantification of CD86-positive cells. Data are presented as means ± SEM (n = 3; ***P < 0.001, one-way ANOVA test with Tukey's multiple comparisons test).

Macrophages activated by ROS are essential in driving the inflammatory response. These cells secrete pro-inflammatory cytokines and chemokines, which attract additional immune cells to the site of damage, thus enhancing the inflammatory milieu 38. Therefore, the clearance of ROS within macrophages and the reduction of inflammation are critical for the remission of ALF. To better understand the biocatalytic ROS elimination properties and anti-inflammatory activities of QFN, we investigated oxidative cell models using the mouse macrophage cell line RAW264.7 cells. Cell viability assays demonstrated that QFN was highly biocompatible in RAW264.7 cells **(Figure S5)**. Additionally, we assessed the ability of RAW264.7 cells to uptake QFN and observed that over 80% of the QFN material was internalized by these cells within 4 h** (Figure S6)**. Intracellular ROS production in Raw264.7 cells was successfully induced by H_2_O_2_, as illustrated by the strong fluorescent intensity of ROS-detecting probes, including DCFH-DA and DHE **(Figure 4A-D)**. Fluorescence images revealed that QFN treatment, similar to quercetin alone, significantly scavenged ROS and mitigated oxidative stress** (Figure 4A and 4C)**. This observation was further supported by the results from flow cytometry analysis using DCFH-DA and DHE** (Figure 4B and 4D)**. Additionally, quercetin and QFN also exhibited substantial ROS scavenging activity in hepatocytes, as evidenced by DCFH-DA fluorescent staining and flow cytometry analysis in AML12 cells **(Figure S7)**. At the same mass concentration, the effective molar concentration of quercetin in QFN was significantly lower than that of free quercetin. However, both demonstrated comparable efficacy in *in vitro* antioxidant and anti-inflammatory assays. These findings indicate that QFN not only enhances the water solubility of quercetin but also improves its anti-inflammatory activity to some extent. Macrophages usually differentiate into the M1 phenotype under ROS stimulation and release pro-inflammatory factors, which can further contribute to hepatocyte injury and aggravate ALF progression 39, 40. LPS was employed to establish an *in vitro* model for M1 polarization in Raw264.7 cells. Immunofluorescence analysis demonstrated that pre-treatment with quercetin and QFN before LPS stimulation markedly attenuated the overexpression of the M1 macrophage marker (CD86) in Raw264.7 cells (**Figure 4E**). These results were further substantiated by flow cytometry, which demonstrated a decreased proportion of CD86-positive Raw264.7 cells following incubation with quercetin and QFN (**Figure 4G**). Additionally, the inflammatory response associated with macrophage polarization can cause further inflammatory damage. Therefore, we assessed the gene expression levels of inflammation-related cytokines in Raw264.7 cells and observed a substantial increase in the expression of proinflammatory cytokines (*Il-1α, Il-1β, and Il-6*) following stimulation with LPS. Notably, treatment with quercetin and QFN significantly reduced the expression of these cytokines (**Figure 4F**). These findings indicate that QFN not only inhibits the polarization of macrophages to the M1 phenotype but also enhances the suppression of the LPS-induced inflammatory response.

### Identification of the anti-senescent and hepatoprotective activities of QFN in hepatocytes

**Figure 5:**
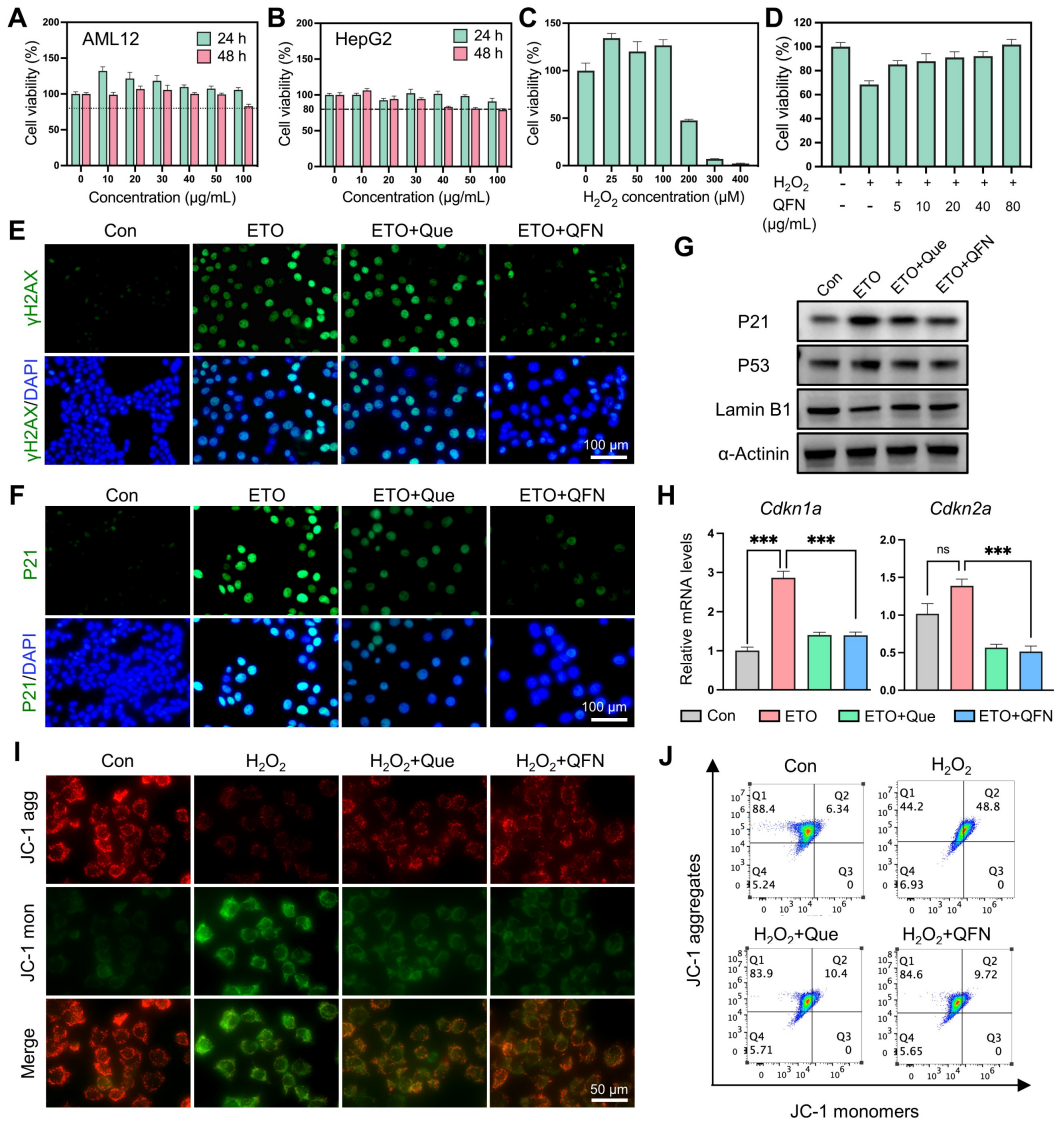
** The anti-senescent and hepatocyte protective effects of QFN *in vitro*. (A-B)** Cell viability of AML12 cells **(A)** and HepG2 cells **(B)** after incubation with different concentrations of QFN for 24 h and 48 h. **(C)** Cell viability of AML12 cells after exposure to indicated concentrations of H_2_O_2_ for 2 h. **(D)** Cell viability of AML12 cells after exposure to 200 μM H_2_O_2_ for 2 h and incubation with different amounts of QFN. **(E-H)** AML12 cells were treated with 4 μM etoposide (ETO) for 24 h and then cultured in an ETO-free medium for another 24 h to induce senescence. Quercetin and QFN were added during the ETO intervention until the cells were collected. Immunofluorescent staining of γH2AX **(E)** and P21 **(F)** in AML12 cells. Scale bar = 100 μm. **(G)** Immunoblot analysis of the protein levels of γH2AX, P53, and Lamin B1. The experiment was repeated three times. **(H)** Gene expression of *Cdkn1a* and *Cdkn2a* (n = 3). Representative images **(I)** and flow cytometry analysis **(J)** of JC-1 in AML12 cells after being stimulated with 100 μM H_2_O_2_ for 2 h which was pretreated with quercetin and QFN for 6 h. Scale bar = 200 μm. Data are presented as means ± SEM (ns: not significant, ***P < 0.001, ****P < 0.0001, one-way ANOVA test with Tukey's multiple comparisons test).

Oxidative stress is a primary driver of aging and cellular senescence 41. Given that QFN can detoxify broad-spectrum ROS and mitigate oxidative damage, it is hypothesized that QFN also possesses protective effects against cellular senescence. First, QFN supplementation exhibited no significant cytotoxic effects on AML12 cells (mouse hepatocytes) and HepG2 cells (human hepatocytes), even at concentrations as high as 100 μg/mL, indicating the good cytocompatibility of QFN **(Figure 5A-B)**. H_2_O_2_ exposure induced severe oxidative stress and significant cell death in AML12 cells, which was markedly reversed by QFN treatment in a concentration-dependent manner, as demonstrated by the cell viability in the CCK8 assay **(Figure 5C-D)**. Furthermore, the live/dead cell double staining assay revealed that both quercetin and QFN effectively counteracted this oxidative stress-induced apoptosis in AML12 cells** (Figure S8)**.

The presence of free radicals can accelerate the aging of neighboring normal cells and impede liver regeneration 7. To explore this further, we used etoposide (ETO), a well-known DNA damage-related senescence inducer, to induce hepatocyte senescence. Previous studies have demonstrated that iron accumulation can induce cellular senescence 42. Since QFN contains iron, we used different concentrations of QFN to stimulate hepatocytes and found that QFN within 40 μg/mL did not cause additional cell senescence **(Figure S9A)**. Additionally, our preliminary experiment used low doses of QFN to intervene in ETO-induced senescent hepatocytes. The results showed that treatment with 10 μg/mL of QFN for 24 h could lead to a decrease in P21 protein levels in senescent AML12 cells** (Figure S9B-C).** Therefore, we chose 10 μg/mL of QFN for subsequent experiments to evaluate the anti-senescent properties of quercetin and QFN. As shown in Figure 5E-F, ETO treatment significantly increased the expression of the DNA damage marker γH2AX and the cell cycle arrest protein P21; however, these effects were largely mitigated by quercetin and QFN, as evidenced by immunofluorescence **(Figure 5E-F)**. The expression of another pivotal regulator of senescence, p53, was also suppressed by quercetin and QFN treatment (**Figure 5G and S10**). The decline in the nuclear membrane integrity protein Lamin B1 caused by ETO was notably restored following QFN treatment, showing results comparable to those with quercetin alone (**Figure 5G and S10**). In addition, analysis of cell cycle arrest genes, including *Cdkn1a* and *Cdkn2a*, further supported the role of QFN in inhibiting cellular senescence** (Figure 5H)**. Furthermore, we also explored the anti-senescent activity of QFN in another human hepatocyte cell line, HepG2, under oxidative stress-induced cellular senescence caused by H_2_O_2_. As illustrated in **Figure S11A**, stimulating HepG2 cells with 300 μM H_2_O_2_ for 1 h, followed by a 3-day recovery period, significantly increased the protein expression levels of P21 and P53** (Figure S11A)**. This result indicates that this method effectively induces cellular senescence in HepG2 cells. The anti-senescent properties of QFN were further confirmed in this model of oxidative stress-induced cellular senescence. Western blot analysis of senescence marker proteins (P21, P53, and Lamin B1) and immunofluorescence staining of γH2AX and P21 consistently demonstrated the protective effects of QFN** (Figure S11B-D)**. Due to the incorporation of Fe elements in the QFN formulation, the molar concentration of QFN at an equivalent mass concentration is lower than that of free quercetin. These findings collectively suggest that the QFN itself may possess intrinsic properties that mitigate oxidative stress and delay cellular senescence, offering promising therapeutic potential for preventing age-related liver dysfunction.

Oxidative stress caused by excessive ROS production leads to mitochondrial dysfunction, which significantly contributes to aging and cell apoptosis 43. Using JC-1, a fluorescent probe for mitochondrial membrane potential (MMP) and early apoptosis, we observed that H_2_O_2_ decreased MMP, as indicated by the reduction of JC-1 aggregates and their transformation into monomers. This detrimental effect was largely mitigated by quercetin and QFN supplementation, as demonstrated by confocal microscopy imaging (**Figure 5I**) and flow cytometry analysis (**Figure 5J**). Since apoptosis and senescence are governed by similar mitochondria-dependent pathways, the observed mitochondrial improvement could concurrently alleviate both cell senescence and apoptosis caused by mitochondrial dysfunction 44. Sublethal mitochondrial apoptotic stress serves as a key trigger for the SASP in aging cells 43. The mitochondrial protective effect of QFN may thus reduce the pro-inflammatory capabilities of these cells by inhibiting the paracrine effects associated with SASP. In summary, these findings suggest that the protective effect of QFN on MMP loss may be a crucial factor underlying its anti-aging and anti-apoptotic properties.

### Biodistribution and biocompatibility analysis of QFN

**Figure 6:**
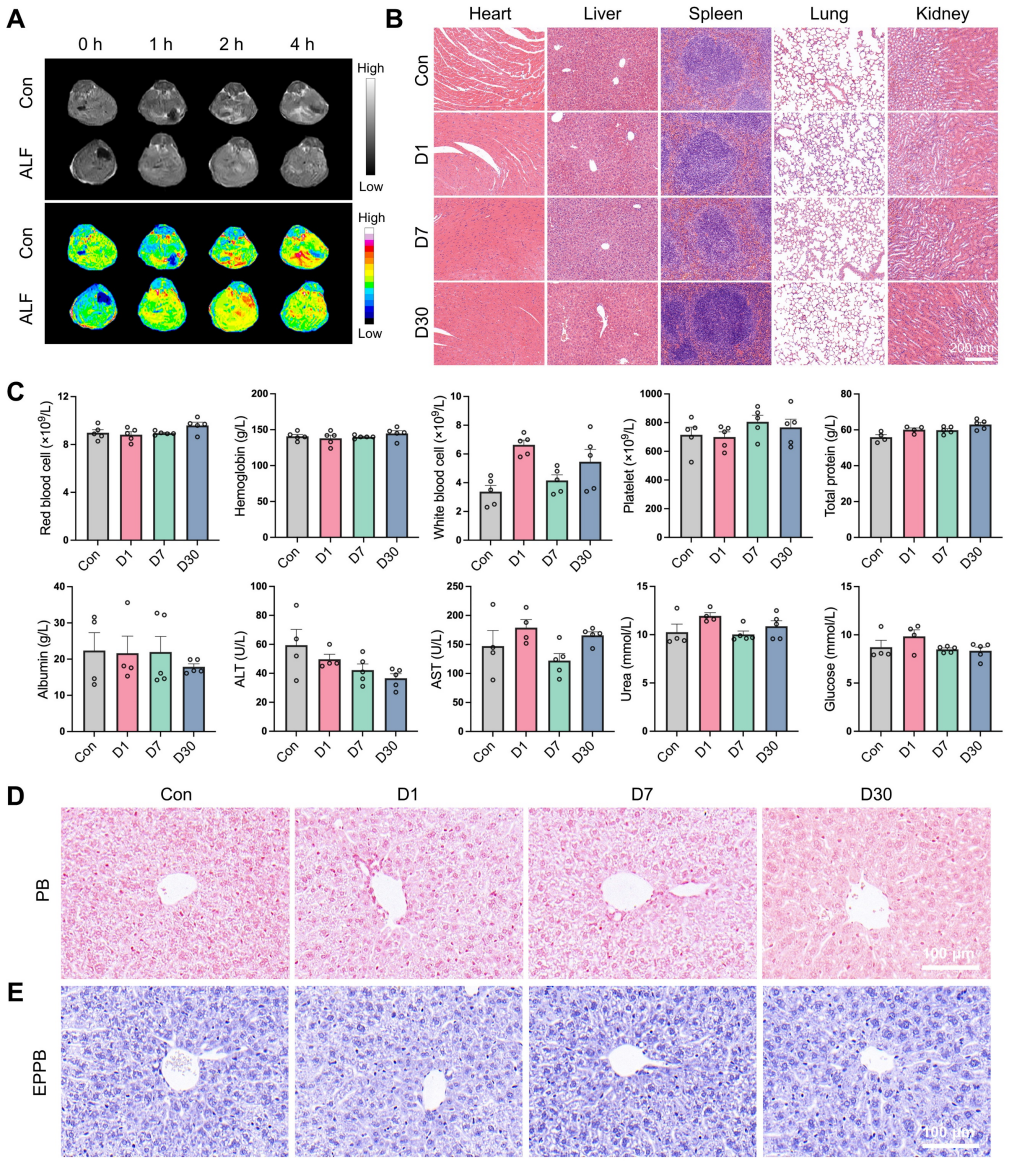
** Biodistribution and biocompatibility analysis of QFN. (A)** Abdominal T1-weighted MR images of C57BL/6J mice at different times after intravenous injection of QFN at 5 mg/kg body weight. Mice were intraperitoneally injected with PBS (Con) or co-administered with 30 μg/kg LPS and 300 mg/kg D-GalN (ALF). **(B-E)** C57BL/6J mice were intravenously injected with QFN at 20 mg/kg body weight and sacrificed 1, 7, and 30 days post-injection (n = 5). **(B)** H&E staining of indicated tissues. Scale bar = 200 μm. **(C)** Complete blood panel analysis of red blood cell, hemoglobin, white blood cell, and platelet, and serum biochemical analysis of total protein, albumin, alanine aminotransferase (ALT), aspartate aminotransferase (AST), glucose, and urea. Data are presented as means ± SEM. Representative images of histochemistry staining for iron by regular Prussian blue (PB) staining **(D)** and DAB-enhanced Perls' Prussian blue (EPPB) staining **(E)** in mouse liver sections. Scale bar = 100 μm.

The excellent anti-inflammatory and anti-senescent properties observed *in vitro* prompted us to explore the potential application of QFN in liver disease, which necessitates liver delivery and good biocompatibility *in vivo*. Based on the T1-weighted MRI signal of QFN demonstrated in previous *in vitro* experiments, we intravenously injected QNF into mice and inspected the biodistribution of QFN by performing T1-weighted MRI scans with an MRI scanner. The results showed that 1 h after intravenous injection, a strong T1 signal was observed in the liver blood vessels, increasing over time, indicating that QFN can rapidly and significantly accumulate in the liver **(Figure 6A)**. QFN also accumulated in the livers of ALF mice, presenting as diffuse T1 hyperintensity, likely caused by extensive hepatocyte death and severe congestion associated with liver failure. In addition, the biodistribution and clearance of QFN were further evaluated using the IVIS spectrum imaging system, which monitored fluorescence intensity changes in major organs of control and ALF mice at multiple time points (10 min, 1 h, 3 h, 6 h, 12 h, and 24 h) following tail vein injection of QFN-Cy5.5. Due to their ultrasmall size, QFN particles rapidly distributed to major organs within 10 min post-injection** (Figure S12A)**. At 1 h post-injection, liver fluorescence intensity in ALF mice was slightly higher than that in control mice, consistent with MRI findings, although the difference was not statistically significant** (Figure S12B)**. Fluorescence intensity in all major organs (heart, liver, spleen, lungs, and kidneys) peaked shortly after injection and gradually declined over time. Notably, over 50% of the QFN-Cy5.5 signal persisted in the liver at 6 h post-injection. Kidney fluorescence dynamics indicated rapid renal clearance, with complete excretion within 24 h, thereby minimizing the risk of long-term accumulation and enhancing overall safety **(Figure S12C-D)**. Interestingly, fluorescence intensity in the lungs remained significantly higher than in other organs across all time points, likely due to the dense capillary network and small microvascular diameter, which may facilitate QFN retention. Collectively, these results demonstrate that QFN efficiently distributes to multiple organs, particularly the liver, and is rapidly cleared via renal excretion, reducing the potential for accumulation-related toxicity and supporting its safety profile for long-term applications.

Moreover, the biosafety of QFN was comprehensively assessed through various methodologies. The hemolytic assay demonstrated that QFN did not induce significant hemolysis, thereby confirming its favorable hemocompatibility **(Figure S13)**. Subsequently, we intravenously injected QFN at a high dose of 20 mg/kg body weight into C57BL/6J mice and collected blood samples, as well as major tissues, at 1, 7 and 30 days post-injection. H&E staining of major organs, including the heart, liver, spleen, lungs, and kidneys, indicated the biocompatibility of QFN (**Figure 6B**). This safety profile was further corroborated by serological tests. Compared with the control group, QFN injection caused negligible differences in routine blood parameters (red blood cells, hemoglobin, white blood cells, and platelets) and serum biochemistry parameters (total protein, albumin, ALT, AST, urea, and glucose) both in the short and long term (**Figure 6C**). Additionally, we assessed iron accumulation in the liver using Perl's Prussian blue staining, both regular and enhanced, and observed no hepatic iron deposition in any of the groups **(Figure 6D-E)**. These findings indicate that QFN exhibits excellent biosafety *in vivo*, making it a promising nanoplatform for the diagnosis and therapy of oxidative stress and inflammation-related diseases.

### Protective efficacy of QFN in a mice model of ALF

**Figure 7:**
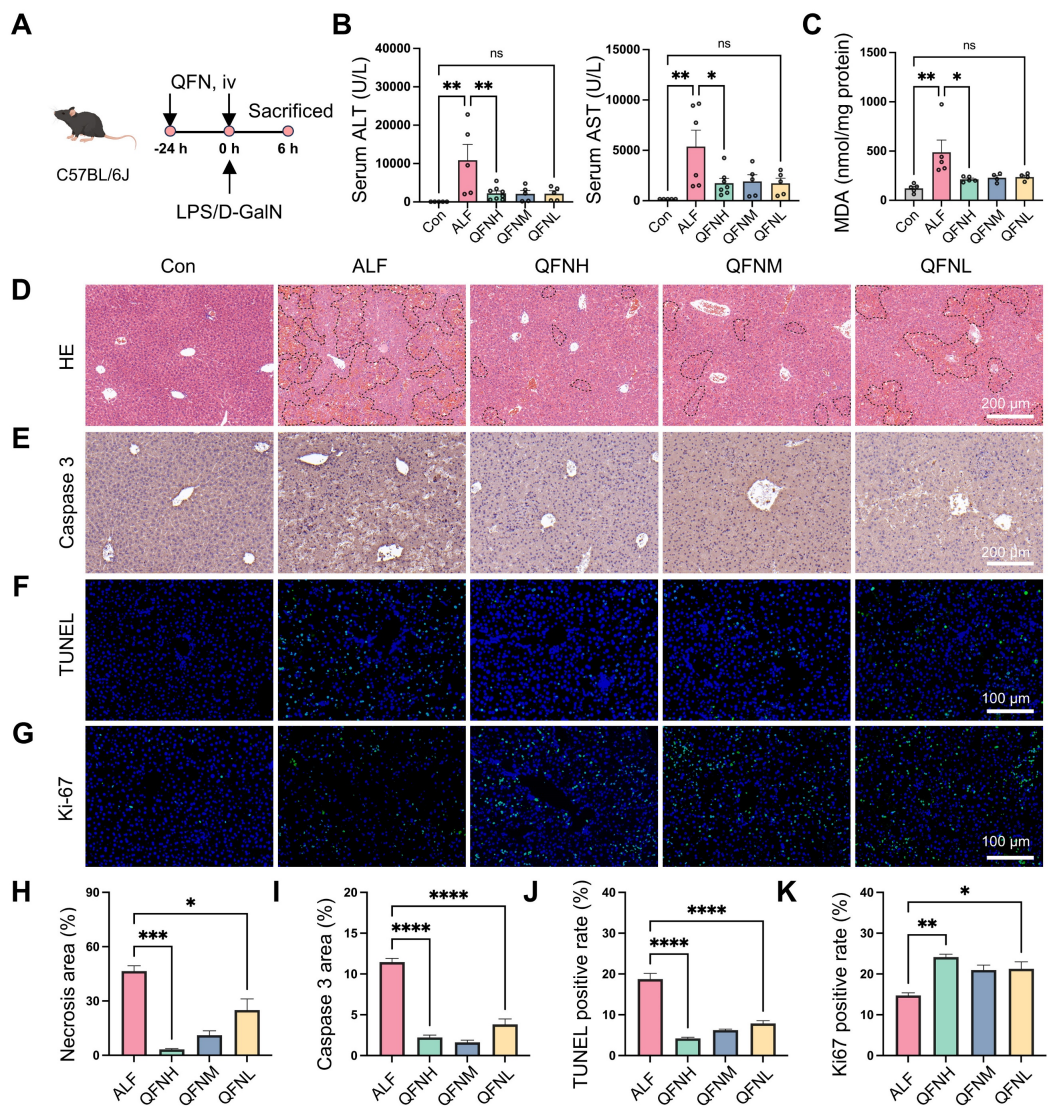
** Hepatoprotective effects of QFN in LPS/D-GalN-induced ALF in mice. (A)** Schematic of ALF induction and treatment regimen, by Figdraw. C57BL/6J mice were intraperitoneally injected with PBS (Con) or co-administered 30 μg/kg LPS and 300 mg/kg D-GalN (ALF). QFN was intravenously injected at high (10 mg/kg body weight), medium (5 mg/kg body weight), and low (2.5 mg/kg body weight) doses 24 h before and concurrently with LPS/D-GalN administration. **(B)** Plasma ALT and AST levels in mice (n = 5-8). **(C)** MDA concentrations in the liver tissues (n = 5 in Con, ALF, and QFNH group; n = 4 in QFNM and QFNL group). **(D)** H&E staining of liver necrosis. Dashed lines delineate the necrotic areas. Scale bar = 200 μm. **(E)** Immunohistochemistry images of Caspase 3 staining in the liver. Scale bar = 200 μm. **(F-G)** Representative immunofluorescence staining of TUNEL (Green) and Ki-67 (Green) in mouse liver sections. Blue: DAPI-labelled cell nuclei. Scale bar = 100 μm. **(H)** The quantitative necrosis area from H&E images (n = 3). **(I)** The quantitative analysis of the positive Caspase 3 area in the liver (n = 3). In the quantitative analysis of the TUNEL-positive rate **(J)** and Ki-67-positive rate **(K)**, the positive cells were counted and normalized to the total number of nuclei detected by DAPI in the liver (n = 3). Data are presented as means ± SEM. (ns: not significant, *P < 0.05, **P < 0.01, ***P < 0.001, ****P < 0.0001, one-way ANOVA test with Tukey's multiple comparisons test).

ALF is characterized by massive ROS production and a severe inflammatory response. Given the ROS scavenging and anti-inflammatory properties of QFN, it may have a protective role in ALF. To explore this potential, we established a lipopolysaccharide (LPS)/D-galactosamine (D-GalN)-induced ALF mouse model. C57BL/6J mice were intravenously injected with various doses of QFN (High: 10 mg/kg body weight; Medium: 5 mg/kg body weight; Low: 2.5 mg/kg body weight) twice: 24 h before and simultaneously with LPS/D-GalN administration (**Figure 7A**). Alanine aminotransferase (ALT) and aspartate aminotransferase (AST), well-established biomarkers of liver function, showed a dramatic increase 6 h after LPS/D-GalN injection, confirming the successful establishment of the ALF model (**Figure 7B**). At all tested doses, QFN treatment significantly lowered ALT and AST levels, indicating its protective role in preventing liver damage (**Figure 7B**). As a key byproduct of lipid peroxidation, malondialdehyde (MDA) levels can serve as an indicator of oxidative stress in patients with ALF 45. We observed that MDA levels were significantly increased in ALF mice but were effectively suppressed by QFN therapy (**Figure 7C**). Moreover, QFN administration significantly decreased necrotic areas in the liver in a dose-dependent manner, as shown by H&E staining (**Figure 7D and 7H**). We also assessed cell apoptosis via immunohistochemistry for Caspase-3 and immunofluorescence staining using TUNEL (terminal deoxynucleotidyl transferase-mediated dUTP nick end labeling). Consistent with serum biomarkers and H&E results, QFN treatment significantly reduced apoptosis in ALF mice, with the highest dose group showing the best hepatoprotective effects (**Figure 7E-F and 7I-J**). These findings were further supported by Ki-67 staining, which revealed a higher number of Ki-67-positive cells in the livers of the QFN-treated group compared to the ALF-challenged group, indicating that QFN promotes cell proliferation and liver regeneration (**Figures 7G and 7K**). Collectively, the above results demonstrate that QFN administration can effectively reduce transaminase levels, prevent extensive hepatocyte death, and promote hepatocyte proliferation, thereby exhibiting strong hepatoprotective properties against LPS/D-GalN-induced damage in a murine ALF model. Nanomaterials offer a wide range of applications in the treatment of acute liver failure 46, including not only the use of antioxidant nanoparticles but also nanomaterial-mediated gene delivery 47-49 and the combination of nanotechnology with stem cell transplantation 50-52. In this study, we utilized bionanomaterials to deliver water-insoluble small molecule drugs, which can be applied not only to liver injury-related diseases but also to other conditions characterized by elevated ROS levels.

### Effect of QFN on cellular senescence and inflammation in a mice model of ALF

**Figure 8:**
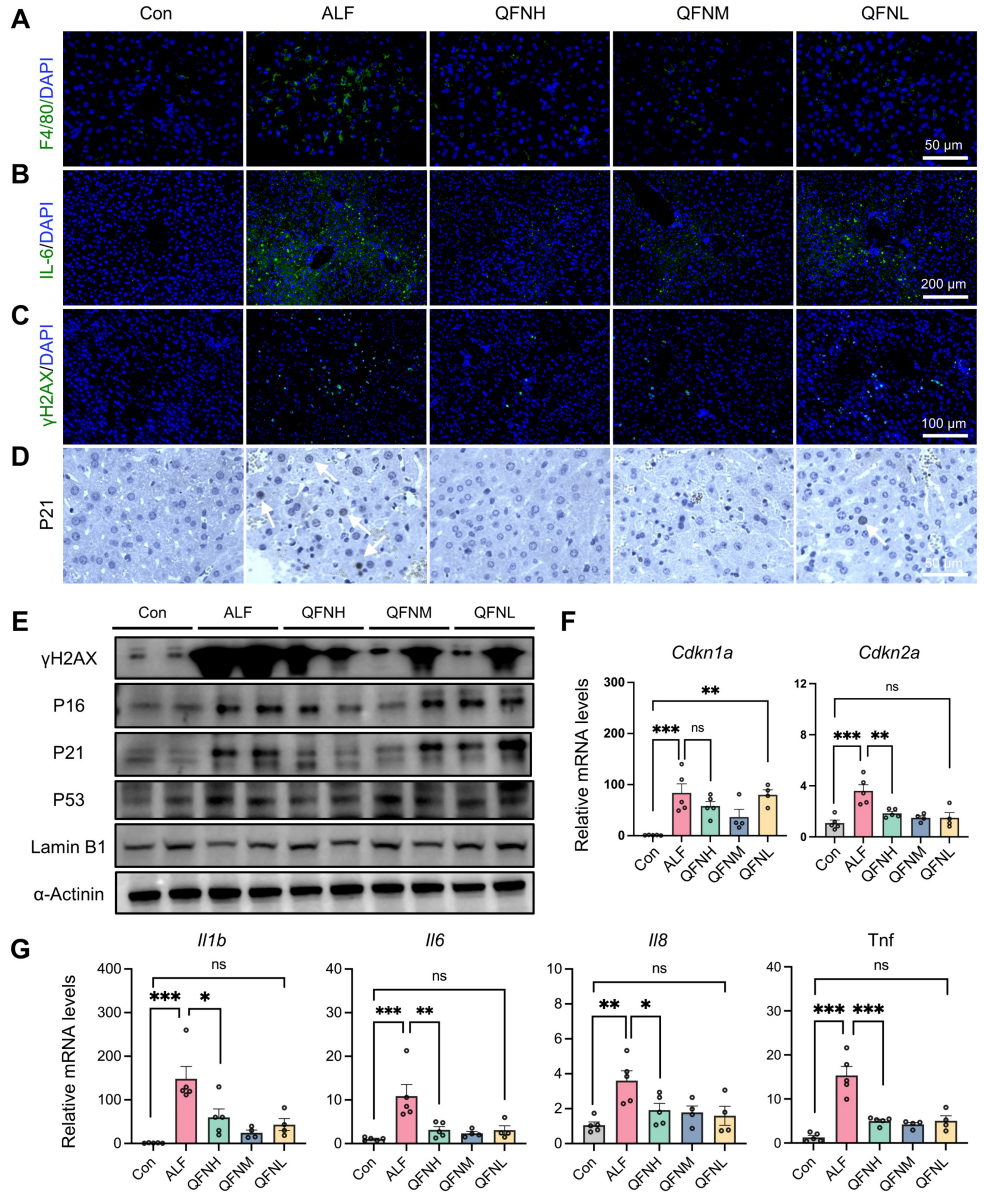
** Protective effects of QFN against cellular senescence and inflammation in ALF mice. (A)** Immunofluorescent images of F4/80 staining in the liver. Scale bar = 50 μm. **(B)** Immunofluorescent images of IL-6 staining in the liver. Scale bar = 200 μm. **(C)** Immunofluorescent images of γH2AX staining in the liver. Scale bar = 100 μm. **(D)** Representative p21 IHC staining in liver sections. The white arrows indicate cells with positive staining. Scale bars = 50 µm. **(E)** Immunoblot analysis of factors related to DNA damage and cellular senescence, including γH2AX, P16, P21, P53, and Lamin B1 in the liver. **(F)** Gene expression of *Cdkn1a* and* Cdkn2a* in the liver tissues (n = 5 in Con, ALF, and QFNH group; n = 4 in QFNM and QFNL group). **(G)** Gene expression of* Il1b, Il6, Il8,* and *Tnf* in the liver tissues (n = 5 in Con, ALF, and QFNH group; n = 4 in QFNM and QFNL group). Data presented as means ± SEM (ns: not significant, *P < 0.05, **P < 0.01, ***P < 0.001, ****P < 0.0001, one-way ANOVA test with Tukey's multiple comparisons test).

Given the role of macrophages in amplifying inflammation and exacerbating hepatic injury through the release of cytokines and chemokines, we used immunofluorescence to detect not only the macrophage marker F4/80 in the liver but also the classical inflammatory marker IL-6 (**Figure 8A-B**). The results showed that QFN injection significantly reduced macrophage infiltration and the inflammatory response in ALF. Hepatocyte senescence induced by liver injury can transmit senescent signals to surrounding cells, impairing hepatocellular regeneration in mice 53. Based on the anti-senescent effects of QFN observed *in vitro*, we further investigated cellular senescence characteristics *in vivo*. Compared to the normal group, LPS/D-GalN-induced ALF mice exhibited classical cellular senescence markers in the liver at both the protein and transcriptome levels (**Figures 8E-G**). Consistent with our *in vitro* observations, QFN injection reduced the expression of the DNA damage marker γH2AX and the cell cycle arrest marker P21 (**Figure 8C-D**). Notably, immunoblot analysis demonstrated that QFN reversed the increase in protein levels of senescence-associated factors, including γH2AX, P16, P21, and P53, in the liver tissue of ALF mice (**Figure 8E**). Additionally, the decrease in Lamin B1, a marker of nuclear membrane integrity observed in ALF, was mitigated by QFN treatment (**Figure 8E**). This anti-senescent ability of QFN *in vivo* was further supported by the reduced transcription levels of genes associated with cellular senescence and SASP. Specifically, the mRNA expression of *Cdkn1a* and *Cdkn2a* was significantly elevated in the liver of the ALF group compared to normal mice, but these levels were greatly reduced following QFN treatment (**Figure 8F**).

In addition, proinflammatory cytokine genes, including *Il1b*,* Il6*, *Il8*, and *Tnf*, were upregulated in the ALF models, but this hepatic inflammatory state was significantly mitigated by QFN administration (**Figure 8G**). These findings suggest that QFN not only mitigates inflammation by reducing macrophage infiltration but also combats cellular senescence, which can decrease SASP expression and further reduce the spread of the inflammatory response. Our experiments have shown that QFN intervention promotes liver regeneration, with its anti-aging properties playing a crucial role in this process. By concurrently inhibiting both inflammation and cellular senescence, QFN disrupts the deleterious cycle in which these processes exacerbate one another, thereby protecting the liver from further damage and fostering a favorable environment for liver regeneration. Consequently, QFN shows promise as a therapeutic option for addressing inflammation and diseases associated with aging.

### Transcriptome analysis reveals the therapeutic mechanism of QFN in ALF

**Figure 9:**
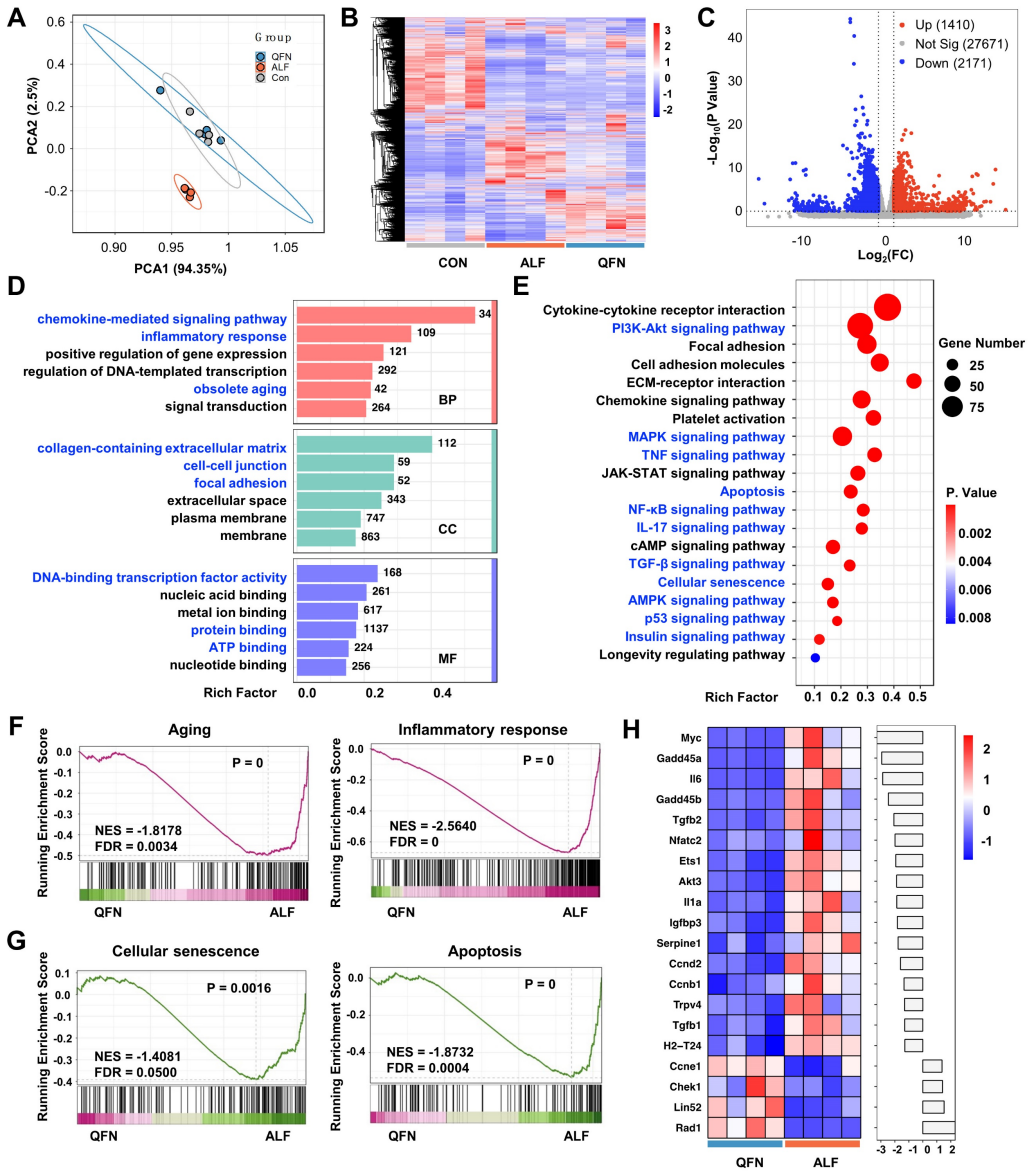
** Transcriptome analysis to reveal the potential biological mechanism.** PCA results **(A)** and heatmap representation and cluster analysis **(B)** for the global RNA-seq genes of control group (Con), acute liver failure group (ALF), and ALF mice treated with QFN at high (10 mg/kg body weight) dose group (QFN). **(C)** Volcano plot visualization of transcriptome gene expression in QFN group vs. ALF Group. Differentially expressed genes (DEGs) including those upregulated (in red) and downregulated ones (in blue) with the greatest significance as determined by the p-value < 0.05 and fold change (FC) > 2. **(D)** Gene ontology (GO) enrichment bar-plot analysis of DEGs in QFN group vs. ALF Group. The DEGs were grouped into three main categories: molecular function (MF), biological process (BP), and cellular component (CC). Each category is organized in ascending order of P values, from smallest to largest (P < 0.05), arranged vertically from top to bottom. The number of genes associated with the specified GO term is depicted on the right of the bar. **(E)** KEGG enrichment scatter plot of DEGs in QFN group vs. ALF Group (P < 0.05). **(F)** GSEA analysis for aging and inflammatory response in QFN group vs. ALF Group. NES, normalized enrichment score. FDR, false discovery rate. **(G)** GSEA analysis for cellular senescence and apoptosis in QFN group vs. ALF Group. NES, normalized enrichment score. FDR, false discovery rate. **(H)** The heatmap bar plot of the top 20 DEGs related to cellular senescence from the QFN group vs. the ALF group was determined by the p-value. The fold change of each gene was log_2_-transformed and subsequently represented as a bar graph.

To further elucidate the therapeutic mechanism of QFN, transcriptome analysis (RNA-sequencing, RNA-seq) was conducted on liver tissues from the control group, the ALF model, and the high-dose QFN-treated group (QFN). Principal component analysis (PCA) revealed distinct clustering of transcriptomes within each group, underscoring significant differences between the QFN and ALF groups. Conversely, the QFN and control groups demonstrated a considerable degree of gene overlap **(Figure 9A)**. This pattern was further corroborated by the heatmap and cluster analysis of the three groups** (Figure 9B)**. In line with previous research, ALF mice exhibited characteristics of cellular senescence and an inflammatory storm. The volcano plot identified 4,443 significantly upregulated genes and 2,805 downregulated genes out of a total of 31,252 genes in ALF mouse liver sections, compared to normal controls** (Figure S14A)**. GSEA revealed that pathways associated with apoptosis, aging, cellular senescence, and inflammatory response were significantly upregulated in the liver of ALF mice **(Figure S14B-E)**. A heatmap was generated to display the top 20 highly expressed genes from a set of 66 cellular senescence-associated genes significantly expressed in ALF mice. These include genes related to cell cycle regulation, such as *Cdkn1a* and *Cdkn2b*, as well as classic SASP members like *Il6, serpine 1, Il-1α, tgfb1, and nfkb1* **(Figure S14F)**. Collectively, these findings suggest that senescence and inflammation are pivotal in the pathogenesis of ALF *in vivo*.

Transcriptome sequencing analysis comparing the QFN group to the ALF group was conducted to explore how QFN modulates the *in situ* microenvironment to confer protection against liver injury. Volcano plots revealed 3,581 significantly differentially expressed genes (DEGs) between the QFN and ALF groups. Of these, 1,410 genes were upregulated, while 2,171 were downregulated **(Figure 9C)**. To further understand the biological functions and pathways of DEGs involved, GO and KEGG pathway enrichment analyses were performed. GO analysis indicated that genes of the QFN group were significantly enriched in biological processes (BP) such as the chemokine-mediated signaling pathway, inflammatory response, and obsolete aging. In terms of cellular components (CC), the DEGs were predominantly associated with the collagen-containing extracellular matrix, cell-cell junction, and focal adhesion. Regarding molecular functions (MF), DEGs were mainly linked to DNA-binding transcription factor activity, protein binding, and ATP binding** (Figure 9D)**.

Consistent with the GO analysis, KEGG pathway analysis further revealed that the DEGs were significantly enriched in pathways related to cellular senescence and inflammation, including p53, PI3K-Akt, NF-κB, IL-17, and TGF-β signaling pathways, all of which are associated with senescence and SASP formation **(Figure 9E)**. Stable cell cycle arrest is a hallmark of senescent cells, with the p53 signaling pathway serving as its primary regulator 54. Beyond its role in halting cell growth, p53 is also crucial for SASP regulation 55. The dual function of p53 in both enforcing cell cycle arrest and controlling SASP underscores its central importance in the biology of cellular senescence and its related inflammatory responses. The PI3K/AKT signaling pathway activates the mTOR pathway, and it is well established that inhibition of mTOR with rapamycin can reduce SASP production and prolong lifespan 56-59. The transcription factor NF-κB is a master regulator of various SASP factors, including interleukins, cytokines, chemokines, and growth factors 60, 61. Aberrant IL-17 signaling in epidermal cells disrupts homeostasis and promotes inflammation, with *in vivo* blockade of IL-17 shown to prevent age-related skin disorders 62, 63. TGF-β, a canonical component of the SASP, regulates the paracrine functions of senescent cells, affecting neighboring cells and inhibiting fracture healing in aged mice 64. Additionally, TGF-β can impair liver regeneration and plays a crucial role in promoting chronic tissue fibrosis through the actions of senescent cells 42, 65. Furthermore, KEGG analysis indicated that additional pathways associated with aging and senescence, including AMPK, MAPK, and insulin signaling pathways, were enriched in the livers of QFN-treated ALF mice 66-68. Additionally, the classical inflammatory TNF-α signaling pathway was also enriched in the livers of QFN-treated ALF mice** (Figure 9E)**. Collectively, our findings suggest that QFN can protect against ALF by modulating various aging and SASP-related signaling pathways to exert anti-aging and anti-inflammatory effects. To further validate these findings, GSEA was performed and demonstrated that senescence pathways were downregulated following QFN treatment. Specifically, QFN therapy was found to reduce aging and cellular senescence, prevent apoptosis, and alleviate the inflammatory response** (Figure 9F-9G)**. The top 20 significantly altered genes related to cellular senescence in the QFN group compared to the ALF group are displayed in the heatmap bar plot, with classical SASP genes, including *Il6, Tgfb2, Il1a, serpine1, and Tgfb1*, showing a significant reduction **(Figure 9H)**. Overall, as revealed by transcriptome analysis, the therapeutic efficacy of QFN in ALF is primarily attributable to its ability to reduce cell apoptosis, inhibit cellular senescence, and alleviate inflammation, thereby promoting the recovery and healing of liver injury.

## Conclusion

In summary, our results suggest that the water solubility of quercetin was significantly improved by its coordination with iron ions, resulting in the formation of a biocompatible ultra-small nanoparticle QFN. The prepared nanoparticles exhibit substantial ROS scavenging ability, targeting both inflammation and cellular senescence, and playing a protective role in acute liver failure. By breaking the vicious cycle between aging and inflammation, QFN can be a promising agent to alleviate oxidative stress-induced inflammatory diseases and aging-related conditions in the future.

## Supplementary Material

Supplementary materials and methods, figures and tables.

## Abbreviations

OH: hydroxyl radicals
ABTS: 2,2'-azino-bis (3-ethylbenzthiazoline-6-sulfonic acid)
ALF: acute liver failure
ALT: alanine aminotransferase
Ami: amiloride
APAP: acetaminophen
AST: aspartate aminotransferase
CPZ: chlorpromazine
DCFH-DA: dichlorodihydrofluorescein diacetate
DEG: differentially expressed genes
D-GalN: D-galactosamine
DHE: dihydroethidium
DPPH: 1,1-diphenyl-2-picrylhydrazyl
ETO: etoposide
FT-IR: Fourier transform infrared spectroscopy
GSEA: Gene Set Enrichment Analysis
ICP-OES: inductively coupled plasma optical emission spectrometry
IL-1β: interleukin-1β
IL-6: interleukin-6
KEGG: the Kyoto Encyclopedia of Genes and Genomes
LPS: lipopolysaccharide
MDA: malondialdehyde
MMP: mitochondrial membrane potential
MβCD: methyl-β-cyclodextrin
MRI: magnetic resonance imaging
NBT: nitro blue tetrazolium
O_2_•^-^: superoxide anion
PCA: principal component analysis
PPI: protein-protein interaction
PVP: polyvinyl pyrrolidone
QFN: quercetin-Fe nanoparticles
Que: quercetin
SASP: senescence-associated secretory phenotype
TCMSP: traditional Chinese medicine systems pharmacology database and analysis platform
TEM: transmission electron microscopy
TGA: thermogravimetric analysis
TMB: tetramethylbenzidine
TNF-α: tumor necrosis factor-α
TUNEL: terminal deoxynucleotidyl transferase-mediated deoxyuridine triphosphate nick end labeling
