# Supplementary material for: Ultra-small quercetin-based nanotherapeutics ameliorate acute liver failure by combatting inflammation/cellular senescence cycle
Source: Theranostics. 2025 Jan 1;15(3):1035–56. doi: 10.7150/thno.103746 (PMC11700872; doi:10.7150/thno.103746)
Supplement: Supplementary file 1 — Supplementary materials and methods, figures and tables. [file thnov15p1035s1.pdf]

Supplementary Information

Ultra-small quercetin-based nanotherapeutics ameliorate acute liver failure by combatting inflammation/cellular senescence cycle

Yali Feng[1,2], Xiaoli Zhang[1,2], Juan Li[1,2], Shan Fu[1,2], Weicheng Xu[1,2], Jinfeng Liu[1,2,3], Yuan Yang[1,2,3], Tianyan Chen[1,2,3], Yingren Zhao[1,2,3], Dongmin Li[4*], Mingzhen Zhang[4*], and Yingli He[1,2,3*]

Affiliations:

1. Department of Infectious Diseases, The First Affiliated Hospital of Xi'an Jiaotong University, Xi'an, Shaanxi, 710061, China
2. Shaanxi Clinical Medical Research Center of Infectious Diseases, Xi'an, Shaanxi, 710061, China
3. National Regional Infectious Diseases Center Co-constructed by National Health Commission of PRC and People's Government of Shaanxi Province, Xi'an, China
4. School of Basic Medical Sciences, Xi'an Jiaotong University, Xi'an, Shaanxi, 710061, China

* Authors for correspondence

Dongmin Li (ORCID: 0000-0002-7808-1109)
Email address: lidongm@mail.xjtu.edu.cn

Mingzhen Zhang (ORCID: 0000-0002-4686-6526)
Email address: mzhang21@xjtu.edu.cn

Yingli He (ORCID: 0000-0002-6439-012X)
Email address: heyingli2000@xjtu.edu.cn

Material and methods

Characterization

The morphology of QFN was characterized using transmission electron microscopy (TEM, Thermo Fisher, USA) operating at 120 kV. The zeta potential of QFN was determined with a Malvern Zetasizer Nano ZS90 Apparatus (Malvern, UK). The UV–Vis absorption spectra of QFN were obtained using an ultraviolet spectrophotometer (Thermo Fisher Scientific, USA), and the FTIR spectra were recorded using a Fourier transform infrared spectrophotometer (Bruker, Germany). Thermogravimetric analysis (TGA) was conducted with a synchronous thermal analyzer (Netzsch, Germany). Absorbance was measured using a microplate reader (BioTek, USA).

ROS scavenging assay: ABTS assay

The total antioxidant capacity of QFN was evaluated using a total antioxidant capacity assay kit based on the 2,2'-azino-bis (3-ethylbenzthiazoline-6-sulfonic acid) (ABTS) method (S0119, Beyotime, China). QFN at various concentrations (5, 10, 20, 30, 40, 50, 100 μg/mL) was mixed with the ABTS radical solution and incubated for 3 min. After incubation, the UV–Vis absorption spectrum of the mixture was recorded, and the absorbance at 734 nm was measured to assess the free radical scavenging activity of QFN. The ABTS•+ scavenging ability was calculated using the following formula: ABTS•+ scavenging ability (%) = $((A\_ABTS - A\_Sample) / A\_ABTS) \times 100\%$, where A_ABTS represents the absorbance of the ABTS solution without treatment, and A_Sample represents the absorbance of the ABTS solution after the addition of QFN.

ROS scavenging assay: DPPH assay

The DPPH (1,1-diphenyl-2-picrylhydrazyl) scavenging assay was employed to assess the antioxidant capacity of QFN. Varying concentrations of QFN (5, 10, 20, 30, 40, 50, and 100 μg/mL) were mixed with a 0.1 mM DPPH radical solution and incubated at room temperature in the absence of light for 30 min. After incubation, the UV–Vis absorption spectrum was recorded, and the absorbance at 517 nm was measured for each group to evaluate QFN's free radical scavenging activity. The DPPH radical scavenging capacity was calculated using the same formula as that used for the ABTS•+ scavenging rate.

Hydroxyl radicals scavenging assay: TMB assay

The Hydroxyl radicals (•OH) scavenging activity of QFN was evaluated using the 3,3',5,5'-tetramethylbenzidine (TMB) method. Different concentrations of QFN (5, 10, 20, 30, 40, 50, and 100 μg/mL) were mixed with a working solution containing 100 μM $FeSO_4$, 500 μM H_2O_2, and 3 mM TMB, followed by incubation in the dark for 10 min. After incubation, the UV–Vis absorption of the oxidized TMB (oxTMB) at 645 nm was measured to assess the •OH scavenging ability of QFN. The •OH clearance rate was calculated using the same formula as the ABTS•+ scavenging rate.

Superoxide anion scavenging assay: NBT assay

The Superoxide anion ($O_2^{\bullet -}$) scavenging activity of QFN was evaluated using the nitroblue tetrazolium (NBT) chromogenic method. Various concentrations of QFN (5, 10, 20, 30, 40, 50, and 100 μg/mL) were mixed with a solution containing 0.05 mM NBT, 13 mM L-methionine, and 20 μM riboflavin in 25 mM PBS buffer (pH 7.4) and exposed to LED light for 5 min. Following illumination, the UV–Vis absorption spectrum was measured at 560 nm using a microplate reader. The $O_2^{\bullet -}$ scavenging rate was

calculated using the same formula as the ABTS•+ scavenging rate. Additionally, the O_2•$^-$ scavenging ability was further assessed by measuring SOD-like activity using a commercial colorimetric SOD assay kit (S311-10, Dojindo Molecular Technologies, Japan) based on the water-soluble tetrazolium (WST) salt method.

In vitro cytotoxicity and cell viability

The cytotoxicity of RAW264.7 cells, AML12 cells, and HepG2 cells was assessed using the CCK-8 method. Cells were plated into 96-well plates and incubated with various concentrations of QFN (10, 20, 30, 40, 50, and 100 μg/mL) for 24 or 48 h. After incubation, cells were incubated with 10% CCK-8 reagent (C6005, NCM Biotech, China) for an hour. Absorbance was then measured spectrophotometrically at 450 nm. Cells that were not treated acted as the negative group, while wells containing only the CCK-8 reagent, without any cells, were used as blanks. Six parallel experiments were performed for each group. Cell viability was determined using the following formula: Cell viability rate (%) = ((OD_test - OD_blank) / (OD_negative control - OD_blank)) × 100%.

Mitochondria and lysosome staining

AML12 cells were cultured overnight and subsequently incubated with 20 μg/mL QFN-Cy5.5 for 6 h. After incubation, the cells were washed with PBS to remove residual QFN from the medium and then stained with Lyso-Tracker Green (C1047, Beyotime, China), Mito-Tracker Green (C1048, Beyotime, China), and Hoechst 33342 (C1022, Beyotime, China). Images were captured using a Zeiss microscope.

Measurement of the mitochondrial membrane potential

The protective ability of QFN was evaluated by assessing changes in mitochondrial membrane

potential (MMP). AML12 cells were cultured overnight, pretreated with 10 μg/mL quercetin or 10 μg/mL QFN for 6 h, and then exposed to 50 μM H_2O_2 for 2 h. MMP levels were measured by staining the cells with 10 μg/mL 5,5’,6,6’-tetrachloro-1,1’,3,3’-tetraethylbenzimidazolocarbocyanine iodide (JC-1, C2005, Beyotime, China) for 15 min. The cells were then washed twice with PBS and analyzed using fluorescence microscopy and flow cytometry.

Hemolysis assay

The hemocompatibility of QFN was assessed using a hemolysis assay. Fresh blood was collected from mice in EDTA-coated tubes, washed three times with PBS (3500 rpm, 5 min, 4°C), and resuspended in ten volumes of PBS. Different concentrations of QFN were dissolved in 0.8 mL PBS and mixed with 0.2 mL of the RBC suspension. PBS and deionized water served as the negative and positive controls, respectively. After incubation at 37 °C for 4 h, the mixture was centrifuged, and the absorbance of the supernatant was measured at 540 nm using a BioTek microplate reader. Each group included three replicates. The hemolysis rate (%) was calculated as follows: Hemolysis rate (%) = ((OD_test - OD_negative control) / (OD_positive control - OD_negative control)) × 100%.

In vivo safety evaluation

Male C57BL/6J mice were randomly assigned to four groups (n = 5). Each group received a single tail vein injection of QFN at a high dose of 20 mg/kg body weight. As a control, an equal volume of saline was injected. Mice were sacrificed at 1, 7, and 30 days after injection. Blood samples were collected to conduct routine blood tests and biochemical analyses. Key organs, including the heart, liver, spleen, lungs, and kidneys, were assessed through H&E staining.

In vivo MRI imaging for biodistribution of QFN

Male C57BL/6J mice were intraperitoneally injected with either PBS (Con) or a combination of 30 μg/kg LPS and 300 mg/kg D-GalN (ALF) group. Following model induction, the mice were immediately administered a 5 mg/kg tail vein injection of QFN. MRI measurements were conducted using a 0.5T magnetic field strength on the NIUMAG MiNiMR60 MRI system (Su Zhou, China). T1-weighted MRI scans were acquired at 0 h, 1 h, 2 h, and 4 h post-injection. The resulting MRI images were processed with Image J software for pseudo-coloring and subsequent analysis.

Ex vivo fluorescent imaging for biodistribution of QFN-Cy5.5

Male C57BL/6J mice were randomly assigned to receive either PBS (Con) or a combination of 30 μg/kg LPS and 300 mg/kg D-GalN (ALF). A 5 mg/kg dose of QFN-Cy5.5 was administered via tail vein injection to both groups. At specified time points post-injection (10 min, 1 h, 3 h, 6 h, 12 h, and 24 h), the mice were euthanized, and major organs, including the heart, liver, spleen, lungs, and kidneys, were collected (n = 3 per time point). Fluorescence imaging of the harvested organs was performed using the IVIS Spectrum Imaging System, and the fluorescent intensity of each organ was quantified for further analysis.

MDA assay

The levels of MDA in whole liver samples were measured with the Lipid Peroxidation MDA Assay Kit (S0131, Beyotime, China), following the manufacturer's protocol precisely, and quantified using a microplate reader. The concentration of MDA was corrected according to the total protein concentration of liver tissue detected by the BCA method.

Western blotting

Cells and liver tissues were lysed using RIPA buffer (WB3100, NCM Biotech, China) supplemented with a protease inhibitor cocktail (P001, NCM Biotech, China). Protein concentrations were determined using a BCA protein assay kit (WB6501, NCM Biotech, China). Equal amounts of protein were then separated by SDS-PAGE and transferred onto PVDF membranes. The membranes were blocked with 5% milk in TBST for 1 h, followed by incubation with primary antibodies overnight at 4 °C. After an hour of incubation with secondary antibodies, the membranes were visualized using an enhanced chemiluminescence system, and the images were quantified using ImageJ software. Details of the antibodies and their dilutions are provided in Supplementary Table 2.

Real-time PCR

Total RNA was isolated from cells and liver tissues using an RNA Isolation Kit with Spin Column (R0027, Beyotime, Shanghai, China), and subsequently reverse transcribed using a reverse transcription kit (RK20428, Abclonal, Wuhan, China). Quantitative Real-Time PCR (qPCR) was performed using SYBR Green Master Mix (RK21203, Abclonal, Wuhan, China) in a Real-Time PCR System. GAPDH was employed as the housekeeping gene, and relative gene expression was calculated using the 2-ΔΔCT method. The primer sequences are listed in Supplementary Table 1.

Histochemical staining

Tissues were fixed with 4% paraformaldehyde and embedded in paraffin. For hematoxylin and eosin (H&E) staining, the tissues were sectioned into 5 μm-thick slices and stained with H&E (G1120, Solarbio, China). Hepatic iron deposition was assessed using the Prussian Blue Iron Stain Kit with

Nuclear Fast Red (G1422, Solarbio, China) and the Prussian Blue Iron Stain Kit with DAB enhancement (G1429, Solarbio, China). The sections were examined under a microscope (Olympus, Japan).

Immunofluorescent staining

Cells fixed in 4% paraformaldehyde and frozen liver sections were used for immunofluorescent staining. The cells and sections were permeabilized with ice-cold PBS containing 0.5% Triton X-100 for 15 min and then blocked with 3% bovine serum albumin (BSA) in PBS for 30 min. Immunostaining with the indicated antibodies was performed overnight at 4°C. The following day, the corresponding secondary antibodies were added, and DAPI was used to stain the nuclei. Images were acquired using a Zeiss microscope. Colocalization analysis was performed using ImageJ software. The antibodies used in this study are listed in Supplementary Table 2.

Immunohistochemistry staining

For immunohistochemistry (IHC) staining, paraffin-embedded liver tissues were sectioned into 5 µm-thick slices. The anti-rabbit immunohistochemical detection kit (PK10009, Proteintech, China) was used for IHC staining. Immunostaining with the indicated antibodies was performed overnight at 4°C. The antibodies and their respective dilutions used in this experiment are listed in Supplementary Table 2.

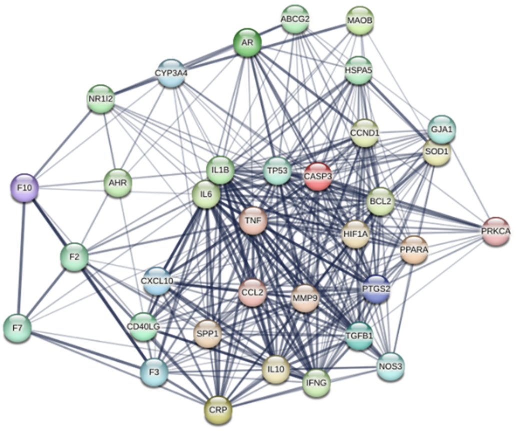

Figure S1. The protein-protein interaction networks were constructed by STRING from shared targets of quercetin and ALF.

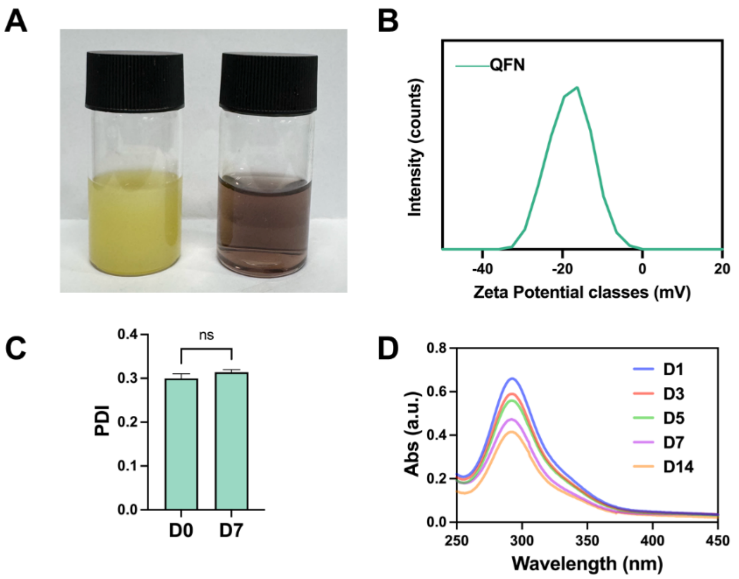

Figure S2. Solubility and stability of QFN. (A) Representative photographs of quercetin (left) and QFN (right) dispersed in ultrapure water. (B) Zeta potential of QFN. (C) The polydispersity index (PDI) of QFN was monitored after seven days (n = 3). Data are presented as mean ± SEM. (D) UV-Vis absorption spectra of QFN were measured over two weeks.

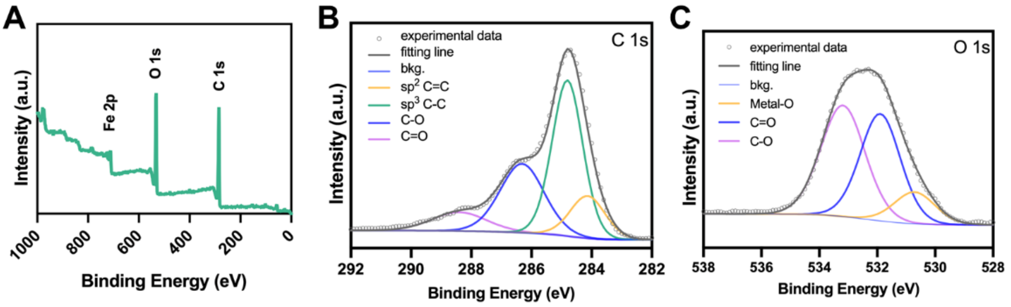

Figure S3. XPS spectra of QFN. (A) X-ray photoelectron spectroscopy (XPS) was employed to analyze the surface elements of the product. Peaks of C elements (B) and O elements (C) are depicted.

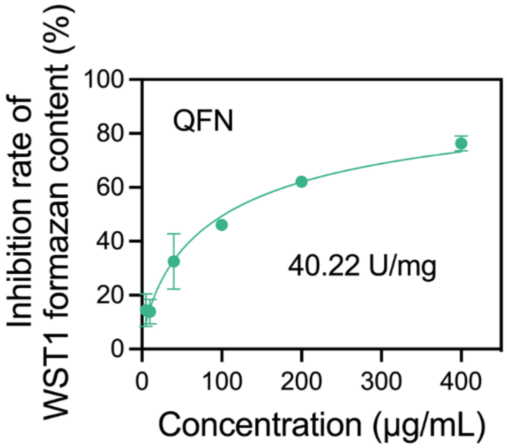

Figure S4. The SOD-like activities of QFN.

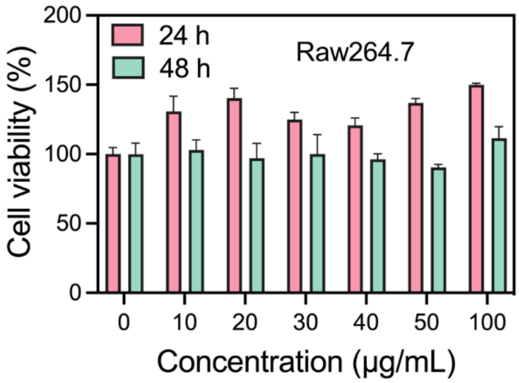

Figure S5. The biocompatibility of QFN in macrophages. Cell viability of Raw264.7 cells after incubation with different concentrations of QFN for 24 h and 48 h. Data are presented as the mean ± SEM (n = 4).

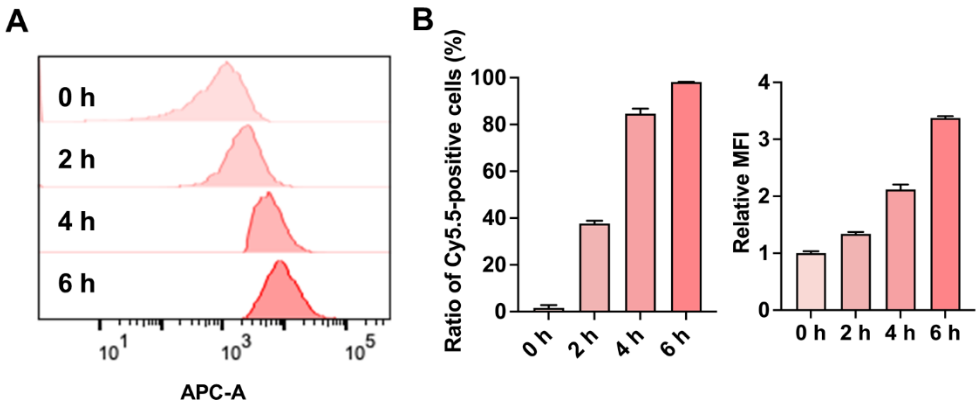

Figure S6. Cellular uptake of QFN in macrophages. (A) The flow cytometry of Raw264.7 cells after incubation with QFN-Cy5.5 for different times. (B) The ratio of Cy5.5-positive Raw264.7 cells (left) and the relative mean fluorescence intensity (MFI) (right) of Cy5.5.

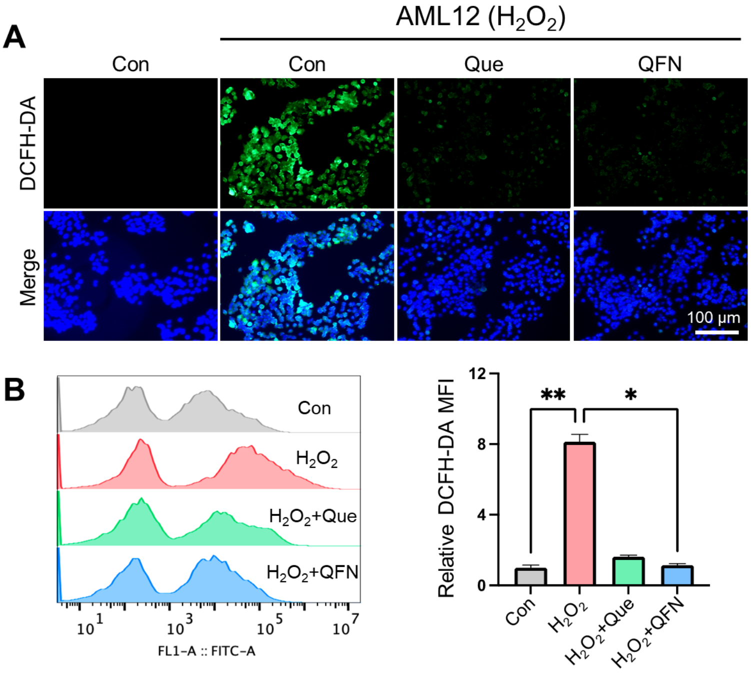

Figure S7. ROS elimination ability of QFN in AML12 cells. (A-B) AML12 cells were pretreated with 10 μg/mL quercetin or 10 μg/mL QFN for 6 h, followed by incubation with 50 μM H_2O_2 for 2 h. (A) Representative images of DCFH-DA staining. Scale bar = 100 μm. (B) Flow cytometry analysis (left) and corresponding relative mean fluorescence intensity (MFI) (right) of DCFH-DA. Data are presented as the mean ± SEM. $^*P < 0.05$, $^{**}P < 0.01$ (one-way ANOVA test with Tukey's multiple comparisons test).

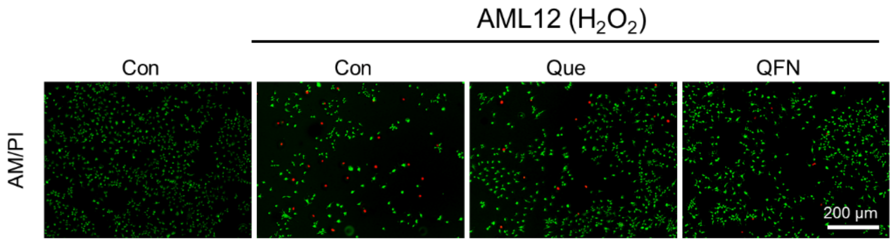

Figure S8. The hepatoprotective effect of QFN. Fluorescence images of AML-12 cells stained with Calcein-AM and propidium iodide (PI) after exposure to 200 μM H_2O_2 for 2 h, followed by incubation with 10 μg/mL quercetin or 10 μg/mL QFN for 24 h.

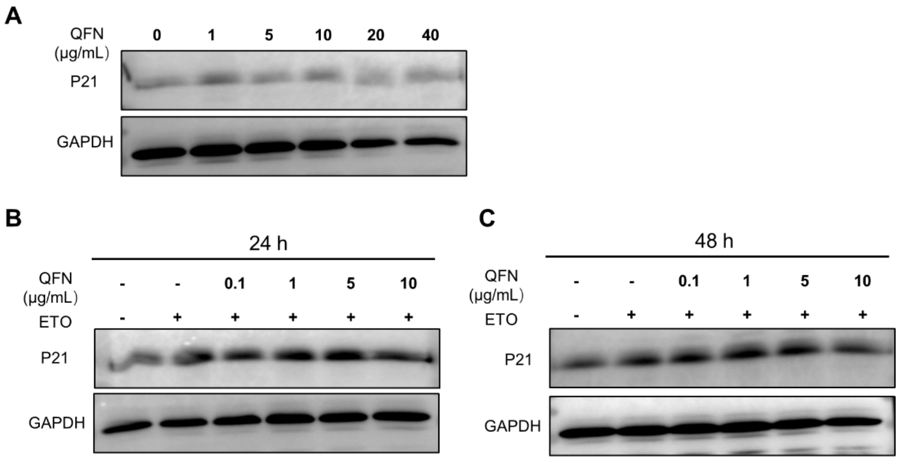

Figure S9. The anti-senescent effect of QFN in AML12 cells. (A) Immunoblot analysis of P21 in AML12 cells treated with virous concentrations of QFN for 24 h. (B-C) Immunoblot analysis of P21 in senescent AML12 cells. AML12 cells were treated with 4 μM etoposide (ETO) for 24 h and then cultured with different concentrations of QFN for 24 h (B) or 48 h (C).

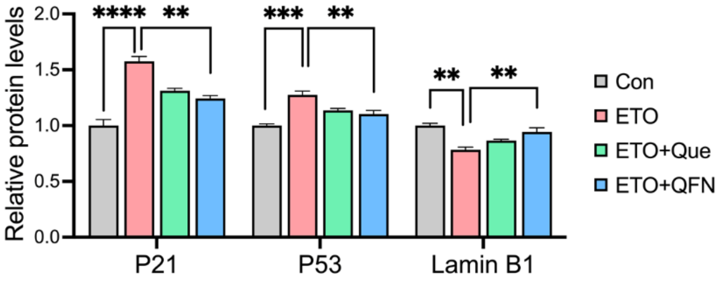

Figure S10. The relative protein levels of P21, P53, and Lamin B1 in AML12 cells were quantified by Image J. This experiment was repeated three times. Data are presented as the mean ± SEM. $^{**}P < 0.01$, $^{***}P < 0.001$, $^{****}P < 0.0001$ (one-way ANOVA test with Tukey’s multiple comparisons test).

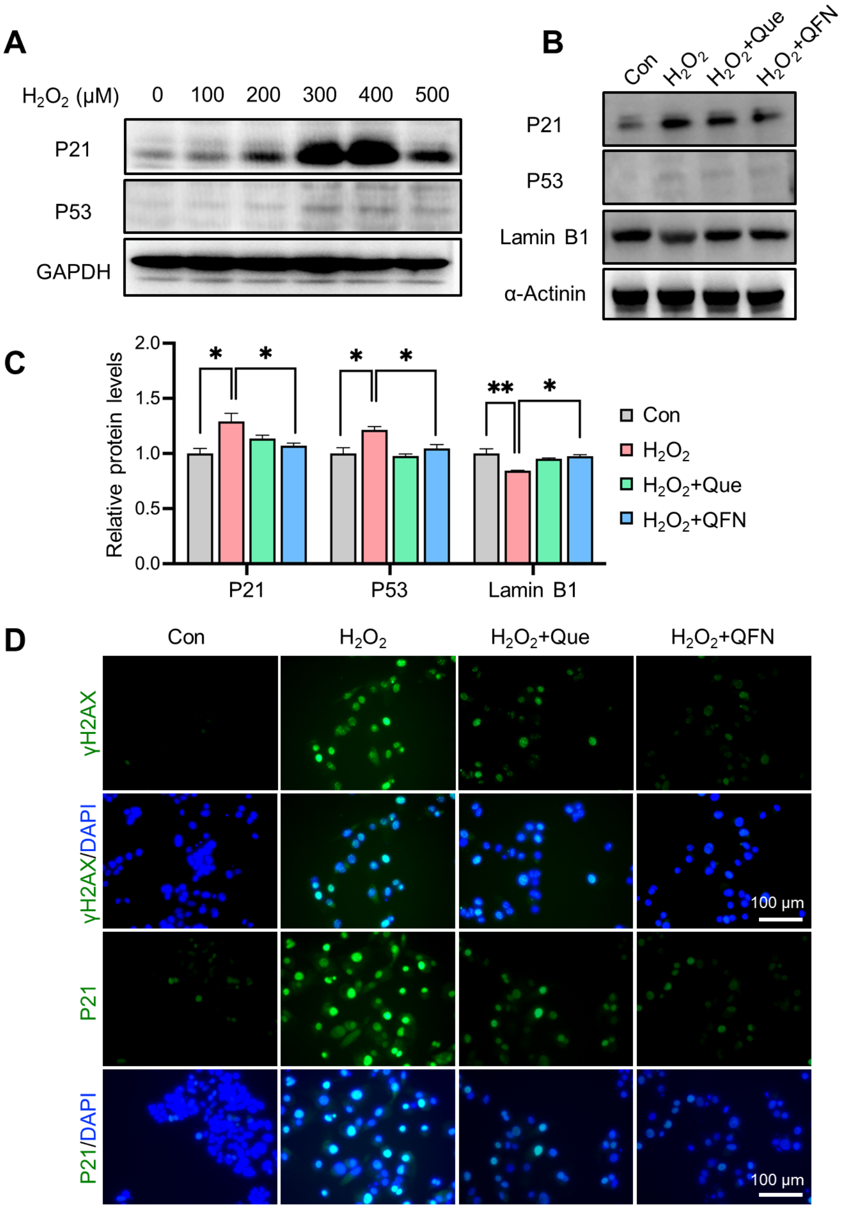

Figure S11. The anti-senescent effect of QFN in an oxidative stress-induced cellular senescence model. (A) HepG2 cells were treated with different concentrations of H_2O_2 for 1 h and then cultured in an H_2O_2-free medium for 3 days. Immunoblot analysis of P21 and P53. (B-C) HepG2 cells were treated with 300 μM H_2O_2 for 1 h and then cultured in an H_2O_2-free medium for 3 days to induce senescence. Quercetin and QFN were added during the recovery period until the cells were collected. Immunoblot analysis (B) and the relative protein levels (C) of P21, P53, and Lamin B1. The experiment was repeated three times. (D) Immunofluorescent staining of γH2AX and P21. Scale bar = 100 μm.

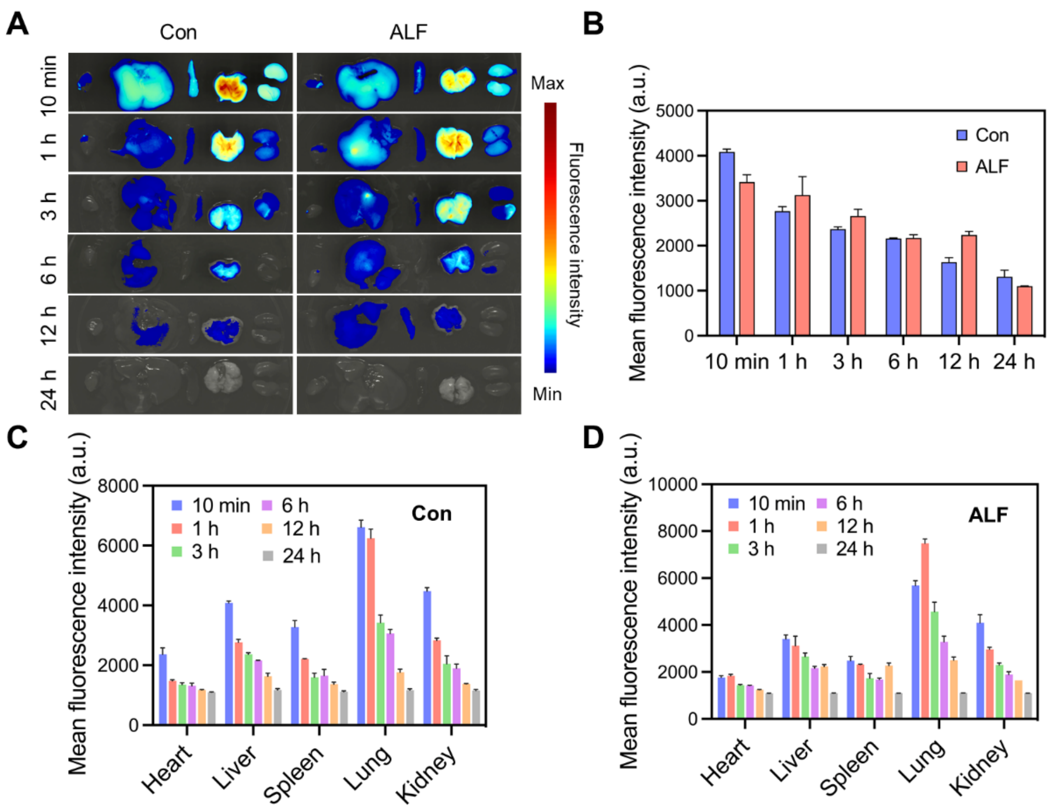

Figure S12. Biodistribution of QFN in control and acute liver failure mice. QFN-Cy5.5 (5 mg/kg) was intravenously injected into both control (Con) and acute liver failure (ALF) mice. At various time points (10 min, 1 h, 3 h, 6 h, 12 h, and 24 h) after injection, the mice were sacrificed, and their heart, liver, spleen, lungs, and kidneys were imaged using the IVIS Spectrum Imaging System (n = 3 per group). (A) Representative ex vivo fluorescent imaging of major organs (from left to right: heart, liver, spleen, lungs, and kidneys). (B) Quantitative analysis of fluorescence intensities in the liver over time in Con and ALF mice. Quantitative analysis of fluorescence intensities of ex vivo organs in the control (C) and ALF (D) group.

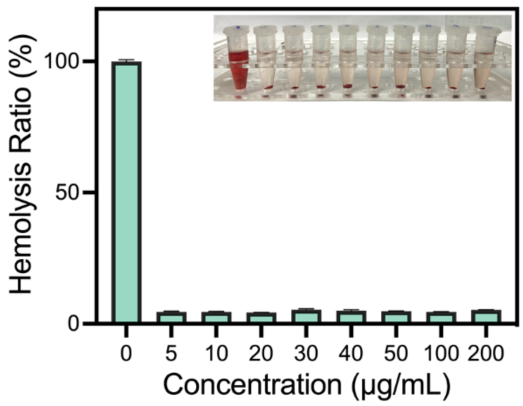

Figure S13. Hemolysis rate of QFN at different concentrations.

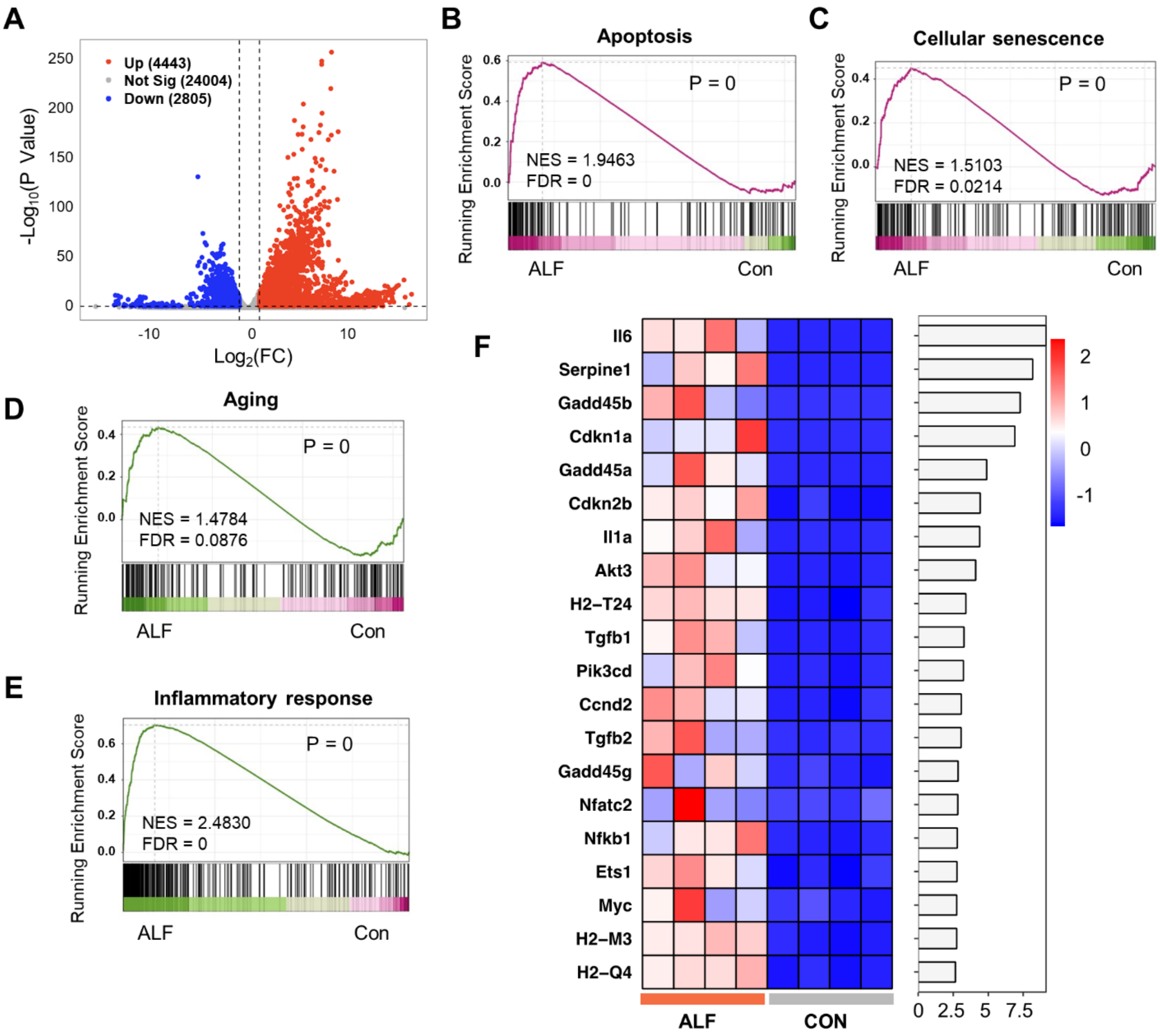

Figure S14. Transcriptomic changes in the liver of mice with ALF. (A) Volcano plot visualization of transcriptome gene expression in the ALF group vs. the Con group. Differentially expressed genes (DEGs) are highlighted, with upregulated genes in red and downregulated genes in blue, identified by p-value < 0.05 and fold change (FC) > 2. Gene Set Enrichment Analysis (GSEA) for apoptosis (B), cellular senescence (C), aging (D), and inflammatory response (E) in the ALF group vs. Con group. NES, normalized enrichment score. FDR, false discovery rate. (F) Heatmap and bar plot of the top 20 DEGs related to cellular senescence in the ALF group vs. Con group, ranked by p-value. The fold change of each gene is $\log_2$-transformed and represented as a bar graph.

Table S1. Primers used for qPCR to detect gene expression in mice

Gene symbol	Primer Forward	Primer Reverse
Cdkn1a	CCTTGTCGCTGTCTTGCACTCTG	GCTGGTCTGCCTCCGTTTTCG
Cdkn2a	TTCAGGTGATGATGATGGGCAACG	CGGGCGGGAGAAGGTAGTGG
IL-1a	GTATGCCTACTCGTCGGGAGGAG	GCAACACGGGCTGGTCTTCTC
IL-1b	CACTACAGGCTCCGAGATGAACAAC	TGTCGTTGCTTGGTTCTCCTTGTAC
Il6	CTTCTTGGGACTGATGCTGGTGAC	TCTGTTGGGAGTGGTATCCTCTGTG
IL8	GCTGCTCAAGGCTGGTCCATG	CATCGTAGCTCTTGAGTGTCACAGG
Tnf	CGCTCTTCTGTCTACTGAACTTCGG	GTGGTTTGTGAGTGTGAGGGTCTG

Table S2. The primary antibody used for immunoblot, IF, and IHC

Antibody	Host Animal	Dilution for Immunoblot	Dilution for IF	Dilution for IHC	Distributor	Cat. Num.
Caspase 3	Rabbit	-	-	1:200	Servicebio	GB11009
F4/80	Rabbit	-	1:500	-	Servicebio	GB11027
IL-6	Rabbit	-	1:500	-	Servicebio	GB11117
Ki-67	Mouse	-	1:200	-	Servicebio	GB121141
Lamin B1	Mouse	1:20000	-	-	Proteintech	66095-1-Ig
P16	Rabbit	1:2000	-	-	Abcam	ab51243
P21	Rabbit	1:2000	1:500	1:400	Abcam	ab188224
P21	Rabbit	1:2000	1:1000	-	Abcam	ab109520
P53	Mouse	1:1000	-	-	Cell signaling technology	#2524
α-Actinin	Rabbit	1:1000	-	-	Cell signaling technology	#6487
γH2AX	Rabbit	1:1000	1:300	-	Cell signaling technology	#9718
